# Mapping immune responses to TAK-003 dengue vaccine: immunogenicity across the clinical development program

**DOI:** 10.3389/fimmu.2026.1851238

**Published:** 2026-07-22

**Authors:** Sanja Mandaric, Tarek El Hindi, Eduardo J. M. Nascimento, Xavier Saez-Llorens, Charissa Borja-Tabora, Heather Friberg, Vianney Tricou, Nicholas Roubinis, Raphael Gottardo, Jeffrey R. Currier, Mayuri Sharma, Shibadas Biswal

**Affiliations:** 1 Takeda Pharmaceuticals International AG, Zurich, Switzerland; 2 Takeda Vaccines, Inc., Cambridge, MA, United States; 3 Pediatric Infectious Diseases, Hospital del Niño Dr. José Renán Esquivel, Sistema Nacional de Investigación at SENACYT, Centro de Vacunación Internacional (Cevaxin), Panama City, Panama; 4 Clinical Research Division, Research Institute for Tropical Medicine, Muntinlupa, Philippines; 5 Viral Diseases Program, Center for Infectious Disease Research, Walter Reed Army Institute of Research, Silver Spring, MD, United States; 6 Lausanne University Hospital and University of Lausanne, Lausanne, Switzerland

**Keywords:** cellular immunity, dengue, humoral immunity, immunogenicity, neutralising antibodies, TAK-003, T-cells

## Abstract

Despite deployment of vector control and prevention strategies, dengue remains a substantial threat to public health. Development of an effective vaccine against dengue has been challenging due to the need for simultaneous immunity against all four dengue virus serotypes, durable protection regardless of prior dengue exposure, and the complex epidemiological patterns of serotype co-circulation. As part of the clinical development of TAK-003, a live attenuated tetravalent dengue vaccine based on a DENV-2 backbone, extensive profiling of post-vaccination immune responses has been performed in participants who were dengue-naive or previously-exposed at baseline across dengue-endemic and non-endemic regions. In this review, we summarise the magnitude, quality, and functionality of humoral and cellular immune responses following the first and second doses of TAK-003 across age, region, and DENV serotype. We also outline long-term persistence and durability, ongoing investigations into correlates of protection, age-stratified responses, and evidence on co-administration with other vaccines.

## Graphical Abstract

**Figure:**
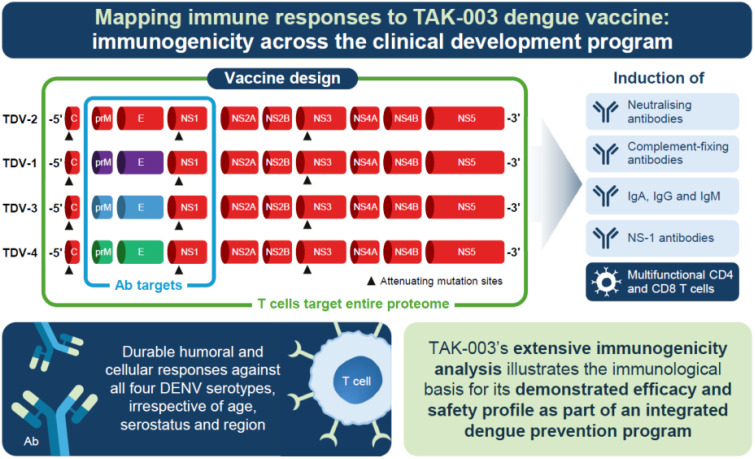

## Introduction

Dengue fever, a mosquito-borne infectious disease caused by dengue virus (DENV), is endemic in over 100 countries worldwide (1). Four closely related but antigenically distinct dengue virus serotypes (DENV-1, DENV-2, DENV-3, and DENV-4) circulate simultaneously, potentially exposing individuals to multiple DENV infections in their lifetimes. Despite global strategies for dengue prevention and control (2, 3), recent years have reported the highest ever rates of dengue (1, 4). While the majority (~75%) of the estimated 390 million dengue infections annually (5) are thought to be asymptomatic or subclinical, approximately 5% of patients with clinical symptoms develop potentially life-threatening symptoms of severe dengue (6).

Dengue immunity is complex and yet to be fully elucidated. Natural DENV infection triggers both innate and adaptive immune responses, with distinct arms of the immune system potentially contributing differently to protection against symptomatic versus severe disease. In addition, unlike many other vaccine-preventable infectious diseases such as measles, pneumococcus, and yellow fever, there is no established immunological correlate of protection for dengue to assess potential vaccine efficacy (7). To date, no single immune marker or combination of immune markers have been identified that fully explains disease risk or protection (8), and due to the complex and multifaceted nature of immune response to dengue, any future correlates of protection may vary by specific serotype, baseline immune status and may involve multiple immune arms (8, 9).

Other challenges to effective dengue vaccine development include the need for immunity against all four DENV serotypes, an incomplete understanding of dengue immunity, and the lack of a disease-relevant animal model (10). While primary infections provide temporary cross-serotype protective immunity and typically lifelong protection against the infecting DENV serotype, secondary infections have been associated with an increased risk of severe dengue, while third and fourth infections often lead to milder outcomes, suggesting exposure to multiple serotypes is needed for protective immunity (11, 12). One possible immunological mechanism underlying severe dengue during secondary infection is antibody-dependent enhancement in which cross-reactive antibodies facilitate viral entry into host cells via Fc receptors and potentially triggering autophagy in macrophages, resulting in weakened early antiviral immune responses (13). However, growing evidence indicates that severe disease also frequently occurs during primary infections, accounting for up to 50% of all severe dengue cases (14). Other contributing factors which play a key role in disease outcomes include underlying comorbidities, available medical care, and specific virus/host factors (15, 16). Additionally, although it has been assumed that infection with an individual DENV serotype leads to life-long protection against re-infection, cases of homotypic reinfection have been reported, which challenges our current understanding of the durability of natural dengue immunity (17, 18). Thus, particularly in the face of increased and variable serotype co-circulation globally, development of tetravalent immunogenicity following vaccination is important to ensure long-term protection against all four serotypes.

Given these complexities, while the WHO recommends serotype-specific neutralising antibody (NAb) titres for evaluating dengue vaccine candidates (19), evaluation of cell-mediated immunity is also encouraged for assessment of immunological memory and duration of protection. Furthermore, short-term antibody kinetics should be evaluated, together with longer follow-up studies of approximately 3–5 years for collection of data on antibody persistence (12, 19). Due to the inherent complexity of dengue immunity, complementary immunological metrices assessing antibody affinity/avidity, isotypes and effector functions, serotype-specific antibodies and depletion assays may help to fully capture the breadth of vaccine immunogenicity (8). In the absence of an established correlate of protection, a systems immunology approach that evaluates multiple dimensions of the immune response, combined with robust statistical analyses, may help assess potential dengue vaccine effectiveness.

TAK-003 (QDENGA ^®^) is a live-attenuated tetravalent dengue vaccine that has been investigated in a comprehensive clinical development program conducted in >28,000 volunteers, of whom ~20,200 received TAK-003, in both dengue non-endemic and endemic areas, in participants from 1.5 up to 60 years of age (20). In its pivotal phase 3 efficacy study (DEN-301, NCT02747927) conducted in children and adolescents living in dengue-endemic regions of Asia and Latin America, TAK-003 demonstrated vaccine efficacy (VE) against virologically confirmed dengue (VCD) of 80.2% (95% confidence interval [95% CI], 73.3–85.3%) after 1-year post-vaccination and a long-term efficacy up to 4.5 years against VCD (61.2%; 95% CI, 56.0–65.8%) and hospitalised dengue (84.1%; 95% CI, 77.8 –88.6%) (21–25). As of 2026, approximately 24 million doses of TAK-003 have been administered globally, and vaccine effectiveness has been demonstrated in real-world studies, with 67.5% effectiveness against hospitalised dengue after the first dose in the 2024 outbreak in Brazil (26). TAK-003 is currently recommended by the WHO for use in children aged 6–16 years regardless of baseline serostatus in settings with high dengue transmission intensity (27).

Previously, an integrated analysis of safety data from the TAK-003 clinical development programme has been published (28). To complement this, in this review we collate all the immunogenicity findings from the randomised clinical trials across various settings (age, region and baseline serostatus) performed to date as part of the clinical development of TAK-003. Based on these data, we attempt to interpret the vaccine’s immunogenicity profile including immune response features post-first and second vaccination, long-term persistence, investigations of potential correlates of protection, immune responses by age, and co-administration with other vaccines.

## Methods

This narrative review summarises evidence of TAK-003 immunogenicity from the clinical development program. PubMed was searched in June 2026 using “TAK-003” combined with immunogenicity-related terms (“immunogenicity,” “immune response,” “cell-mediated response,” “cellular,” “humoral,” “antibody,” and “T cells”), without date or language restrictions. Eligible studies evaluated TAK-003 in humans and reported one or more immune outcomes, including neutralising or binding antibody responses, avidity, complement-fixing antibodies, non-structural protein responses, memory B-cell responses, T-cell responses, or exploratory immune correlates. Real-world studies and unpublished sponsor data were excluded. Where multiple publications reported the same study, the most recent data were used and interim reports were excluded if superseded. Conference abstracts were included only when no corresponding manuscript was available. Studies were prioritised according to relevance to the final licensed vaccine formulation and dosing schedule, with pivotal efficacy studies given the greatest weight, followed by long-term follow-up studies, immunobridging analyses, and exploratory mechanistic substudies. Because this was a narrative review, no formal risk-of-bias assessment or quantitative meta-analysis was performed. Findings were synthesised qualitatively according to study design, population characteristics, baseline serostatus, age group, geography, assay platform, and follow-up duration. Where findings were exploratory or based on small substudies, this is indicated in the text.

## Immune responses to dengue infection

Natural DENV infection triggers both innate and adaptive immune responses. As part of the initial immune response, virally-infected macrophages and Langerhans cells in the skin trigger production of inflammatory mediators, including type I interferons (IFNα/β), which limit early viral replication as well as activating and recruiting adaptive immune cells (29). Binding antibodies prevent infection by blocking viral attachment to the cell surface, neutralising the virus by disrupting membrane fusion, and/or mediating antiviral activity through Fc-dependent effector functions, including complement activation (30). The complement system functions as an effector mechanism of the early antibody response by promoting the opsonisation, killing and clearance of virions (31–35). Complement proteins bind to antigen-antibody complexes on virally-infected cells, facilitating cell lysis and clearance. Immunoglobulin (Ig)M and IgA antibody isotypes are also induced early upon infection and induce virus neutralisation (36–38) and phagocytosis (39).

One of the components of DENV-triggered adaptive immunity are NAbs, which play an important role in the neutralisation of flavivirus infections by blocking viral entry into host cells and are associated with reduced risk of developing symptomatic disease (31, 40). T cell responses also play an established role against DENV by killing virus-infected cells and activating other immune cells (41). In addition, longitudinal cohort studies have shown that cytokine-producing multifunctional T cell responses are associated with protection from symptomatic secondary DENV infection (42). DENV-specific CD4+ T cells target structural (capsid [C], envelope [E]) and non-structural (NS1, NS3, NS5) proteins, mediating cytokine-driven responses that aid in antibody maturation, and in some cases can also exhibit cytotoxicity (43–45). DENV-specific CD8+ T cells primarily target non-structural proteins (e.g., NS3, NS5), exhibiting cytotoxic effects and producing antiviral cytokines crucial for viral control (46). Following primary DENV infection, serotype-specific memory B cells (MBCs) and T cells develop, with accumulation of IgA+, IgG+ and IgM+-specific MBCs over time following multiple exposures to DENV (47). Although durable serotype-specific immunity develops following DENV infection, cross-serotype protection against other DENV serotypes is typically temporary and wanes over months to a few years, contributing to a potential increased risk for severe outcomes from secondary heterologous infections (48).

### Disease severity

One of the hallmark symptoms of severe dengue is increased vascular permeability leading to plasma leakage and, in some cases, shock; thought to arise largely from immune-mediated functional disruption of endothelial barrier integrity. Although there is no correlate of protection for dengue, different arms of the immune response may contribute to protection against severe dengue, including both MBCs and T cells, which perform key functions in long-term persistence and recall responses (49–54). However, the evidence linking specific immune features to protection from severe disease remains limited. Furthermore, NS1 is a viral protein that is secreted into the serum of infected individuals during acute dengue infection, and has been implicated in vascular leakage (55, 56). Antibody responses to NS1 have been shown to be higher in patients with milder dengue symptoms, suggesting an inverse relationship between NS1 antibody titres and vascular leakage (57). Previous studies have shown that lower titres of DENV-specific binding antibodies are typically found in patients with more severe outcomes and higher titres are seen in patients with milder outcomes (58). In addition to magnitude, the strength of antibody-virus interaction (polyclonal avidity) is also an important factor for determining the quality of the antibody response. Repeated dengue infections have been shown to increase the magnitude, avidity, and neutralisation potency of DENV-specific antibodies relative to those post-primary infection (9). Moreover, dengue-specific T cells may also contribute to protection by accelerating viral control (particularly via CD8+ cytotoxicity and IFN-γ–mediated antiviral activity) and by coordinating effective immunity through CD4+ help, thereby potentially reducing the duration and magnitude of viremia that is often associated with severe outcomes (41, 59).

Due to these complex and multifaceted immune responses, a correlate of protection against dengue infection or disease has not yet been established. More research is required to establish specific correlates of protection, which may vary by specific serotype, baseline immune status and may involve multiple immune arms (8, 9). In particular, evidence directly linking vaccine-induced immune features to prevention of severe disease remains limited, in part because severe outcomes are relatively rare and therefore difficult to assess robustly in clinical studies.

## TAK-003 design and antigenic targets

The majority of known NAb epitopes are found on the DENV E protein, whereas T cell epitopes are distributed across the dengue proteome but are more concentrated within the NS proteins (60, 61). To this end, TAK-003 was designed with the aim of facilitating stimulation of a broad immune response to both structural and NS dengue proteins (62), in order to provide protection from both symptomatic and severe disease manifestations. The vaccine is based on a DENV-2 virus backbone with chimeric viruses representing DENV-1, -3, and -4, created by substituting the pre-membrane (prM) and E proteins with those from each of the other three serotypes (63) (Supplementary **Figure 1**). The DENV structural proteins prM and E, which are unique to each serotype, were included with the intention of simultaneously stimulating B cells to produce antibodies against all four serotypes, and with the aim of providing protection across diverse DENV genotypes (64–66).

**Figure 1:**
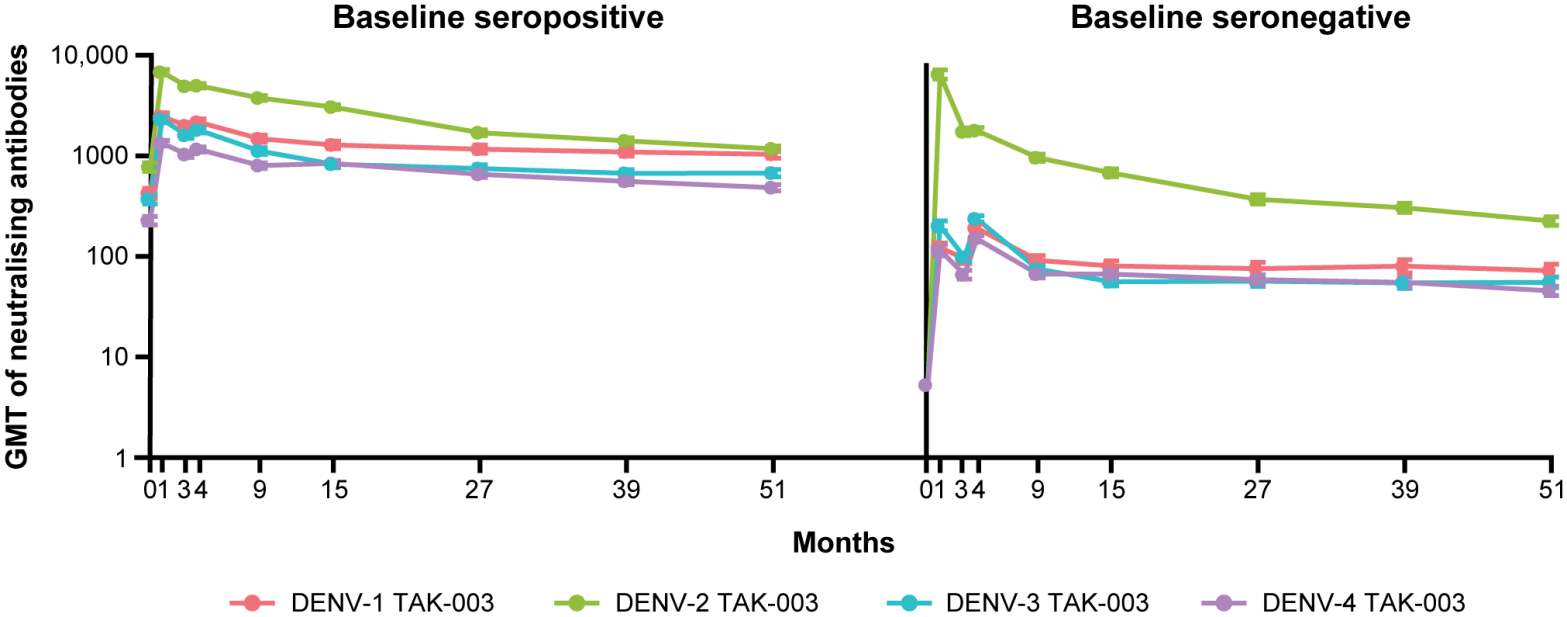
GMTs (95% CI) and b) seropositivity rates of neutralising antibodies in children and adolescents, by dengue serotype and baseline serostatus up to 51 months after first vaccination. CI, confidence interval; GMT, geometric mean titre. Limit of detection: MNT = 10. The terms “seropositive” and “seronegative” refer to baseline dengue serostatus. Seropositivity was defined as a neutralising titre MNT ≥ 10 for at least one DENV serotype. Data from the DEN-301 study which included children aged 4–16 years who received TAK-003 (baseline seropositive n=1816, baseline seronegative n=702). DEN-301 was performed across eight dengue-endemic countries in Asia and Latin America (Brazil, Colombia, Dominican Republic, Nicaragua, Panama, Philippines, Sri Lanka, and Thailand). Reference: *Tricou V*, et al. *Lancet Glob Health. 2024;12(2):e257-e270.*.

The DENV-2 NS1 component of the backbone was included to induce antibodies that bind to DENV-2 NS1 and cross-react with NS1 from other serotypes, with the aim of contributing to protection against severe dengue through blocking viral NS1-mediated vasculopathy (67). The DENV-2 backbone non-structural components (NS1, NS2A, NS2B, NS3, NS4A, NS4B, and NS5) were included with the aim of eliciting multitypic, DENV-specific T cell responses against NS proteins, which may support clearance of infected cells and contribute to durable protection against reinfection and severe disease (62, 64).

## Overview of studies and assays evaluating immunogenicity of TAK-003

As of June 2026, a total of 19 completed clinical trials (21–25, 66–99) were eligible for inclusion in this summary (**Table 1**). Earlier phase 1 and 2 studies assessed immunogenicity of different formulations, administration methods, and schedules of the vaccine, with a final formulation and schedule (2 doses, administered 3 months apart) progressed for further development across 13 phase 2 and 3 studies based on the findings of these earlier studies (67, 71, 76, 83, 84, 86, 87, 89, 91). Phase 1 studies included evaluation of both the subcutaneous and intradermal routes of administration, with the initial idea of replicating the natural route of exposure via mosquito bites. While both routes induced comparable antibody response, the subcutaneous route was found to be preferable due to lower rates of local reactogenicity (84).

**Table 1:** Overview of completed studies contributing to TAK-003 immunogenicity evaluation.

Study number	Phase	Countries	Study summary	Age	Trial period	Immunogenicity endpoints	Total number of participants	No. participants receiving final formulation/schedule included in immunogenicity analyses^†^
Endemic regions								
DEN-203; NCT01511250 (67–71)	2	Colombia, Puerto Rico, Singapore, Thailand	RCT in adults and children	1.5–45 years	November 2011 to April 2016	NAbs, binding antibodies, avidity, CFA, NS1	148 TAK-003: 90 Placebo: 58	0
DEN-204; NCT02302066 (71–75)	2	Dominican Republic, Panama, Philippines	RCT evaluating different vaccine schedules	2–17 years	December 2014 to March 2019	NAbs, binding antibodies, avidity, T cells	1800 TAK-003: 1601 Placebo: 199	83
DEN-205; NCT02425098 (66, 76)	2	Singapore	Randomized study evaluating two different vaccine formulations	18–60 years	June 2015 to September 2017	NAbs, T cells, memory B cells	351 TAK-003: 351	174
DEN-301; NCT02747927 (21–25, 77–80)	3	Brazil, Colombia, Dominican Republic, Nicaragua, Panama, Philippines, Sri Lanka, Thailand	Pivotal efficacy RCT	4–16 years	April 2016 to June 2024	NAbs, TS and CR NAbs, binding antibodies, avidity, CFA, NS1	20,099 TAK-003: 13,401 Placebo: 6698	2518
DEN-308; NCT04313244 (81)	3	Thailand	RCT evaluating coadministration with HPV vaccine	9–14 years	May 2021 to July 2022	NAbs	614 TAK-003 +HPV: 307 HPV: 307	0^¶^
DEN-313; NCT02948829 (82)	2	Panama, Philippines	Open-label study focusing on cell-mediated immunity	4–16 years	April 2017 to December 2020	NAbs, T cells	200 TAK-003: 200	195
Non-endemic regions								
DEN-101; NCT01110551 (83, 84)	1	USA	RCT evaluating subcutaneous vs intradermal administration of low or high dose	18–45 years	June 2010 to April 2012	NAbs, T cells	72 TAK-003: 48 Placebo: 24	0
DEN-102; NCT01224639 (85, 86)	1	Colombia (non-endemic region)	RCT evaluating dose-escalation	18–45 years	October 2010 to November 2011	NAbs, type 1 interferon	96 TAK-003: 79 Placebo: 17	0
DEN-103; NCT01765426 (87)	1	USA	Randomized study evaluating different regimens and methods of administration	18–45 years	February 2013 to June 2014	NAbs	67 TAK-003: 67	0
DEN-104; NCT01542632 (88, 89)	1	USA	Randomized and open-label dose-finding study	18–45 years	January 2012 to January 2014	NAbs	140 TAK-004: 140	0
DEN-105; NCT04964258 (62, 90)	1b	USA	Randomized open-label study evaluating subcutaneous vs intramuscular administration	18–45 years	January 2013 to November 2013	NAbs, T cells	60 TAK-003: 60	0
DEN-106; NCT02193087 (91)	2	USA	Randomized study comparing lyophilised and liquid formulations	18–49 years	July 2014 to May 2015	NAbs	1002 TAK-003: 1002	0
DEN-210; NCT03746015 (92)	2	USA	Open-label study evaluating humoral and T cell responses in flavivirus-naïve and dengue-immune adults	18–60 years	December 2018 to March 2021	NAbs, T cells	30 TAK-003: 30	21
DEN-303; NCT03999996 (93, 94)	3	USA, Mexico City	Long-term persistence in participants from DEN-304 and DEN-315 studies plus booster administration^†^	13–63 years	November 2019 to May 2024	NAbs, T cells*	246	50
DEN-304; NCT03423173 (95)	3	USA	RCT evaluating lot-to-lot consistency	18–60 years	February 2018 to January 2019	NAbs	923 TAK-003: 791 Placebo: 132	447
DEN-305; NCT03342898 (96)	3	USA	RCT evaluating coadministration with yellow fever vaccine	18–60 years	February 2018 to May 2019	NAbs	900 TAK-003: 300 YF: 300 YF + TAK-003: 300	198
DEN-307; NCT03771963 (97)	3	USA	Open-label study evaluating end-of-shelf-life vaccine	18–60 years	March 2019 to March 2020	NAbs	200 TAK-003: 200	139
DEN-314; NCT03525119 (98)	3	UK	RCT evaluating coadministration with hepatitis A vaccine	18–60 years	May 2018 to July 2019	NAbs	900 TAK-003: 300 TAK-003+HAV: 596	208
DEN-315; NCT03341637 (99)	3	Mexico City	RCT in seronegative adolescents	12–17 years	December 2017 to January 2019	NAbs	400 TAK-003: 300 Placebo: 100	271

HAV, hepatitis A vaccine; HPV, human papillomavirus vaccine; NAbs, neutralising antibodies; OLE, open-label extension; RCT, randomised controlled trial; YF, yellow fever.

^*^ analysis of study data ongoing, conference publication available; ^†^Note that phase 1 and some phase 2 studies used an earlier formulation and/or dosing schedule of TAK-003; ^¶^None of the participants received TAK-003 alone.

An additional study (DEN-302; NCT06060067) does not yet have published data available and was not included in this summary.

Another key consideration during early clinical development was the need to optimise the vaccine formulation and balance immune responses to all four serotypes, an important feature as antibody levels required to achieve protection from disease may differ among serotypes (40). The initial TAK-003 formulation showed strongest NAb responses against DENV-2 and lowest against DENV-4; reduction of the TDV-2 component of the vaccine in a later formulation resulted in a more balanced response across the four serotypes (20).

Over the course of TAK-003’s clinical development to date, seven studies have been completed in Latin American and Asian regions considered endemic for dengue (21–25, 66–82) while the rest were completed in USA, UK, Mexico City and Colombia in settings considered non-endemic (62, 83–99). Baseline characteristics across completed studies using the final formulation and dose schedule are shown in **Supplementary Table 1**.

Across all the studies, immunogenicity was evaluated as geometric mean titres (GMTs) of NAbs against each of the four serotypes, measured by a validated dengue microneutralisation test (MNT) and expressed as the reciprocal of the dilution of test serum showing a 50% reduction in plaque counts versus virus-only controls (MNT_50_) (22). MNT is based on the same principles as the plaque reduction neutralisation test (PRNT), the considered “gold standard” assay for detection and quantification of dengue NAbs (100), but with higher throughput allowing testing of a greater number of samples (101), and therefore was used in preference to PRNT throughout TAK-003 clinical development. While providing a standardised approach for measuring NAb titres throughout the clinical development programme, due to differences among the trials in design, objectives, setting, and study populations, including endemic versus non-endemic regions, baseline serostatus distribution, paediatric versus adult subjects, and the timing of immunogenicity assessments, immunogenicity data were not pooled across trials and were summarised descriptively.

Seropositivity was defined as an MNT_50_ titre ≥10 (the limit of detection of the assay) against ≥1 dengue serotype. T cell responses were assessed in children/adolescents from endemic and adults from non-endemic regions using (IFN-γ) enzyme-linked immunospot (ELISPOT) and flow cytometry-based intracellular cytokine staining (ICS) assays (62, 75, 82, 83, 92). Fit-for-purpose exploratory immunogenicity assessments were performed in a subset of participants from endemic (DEN-203, DEN-204, DEN-205, DEN-301) and non-endemic (DEN-102, DEN-304) regions and included avidity of the binding IgG response (70), complement-fixing antibody (CFA) responses (102), NS1 IgG responses (67), IgA and IgM responses (68), MBC responses (66), and assessment of type-specific (TS) and cross-reactive (CR) NAbs (65, 88).

## Early immune responses following the first dose of TAK-003

### Innate immune responses

Two studies have performed small-scale exploratory transcriptional profiling of responses after a first dose of TAK-003. An exploratory phase 1 study in seronegative adults who received an early formulation of TAK-003 in dengue non-endemic areas of Columbia (DEN-102) revealed that TAK-003 vaccination was associated with upregulation of genes related to type I interferon pathways and expansion of immune cells within 7 days post-vaccination (85). A further exploratory study in adults from dengue non-endemic region (DEN-210) receiving the final formulation showed that vaccinated seronegative subjects mounted a transient type I IFN response and upregulation of genes involved in viral sensing after dose 1, whereas seropositive subjects exhibited a distinct largely downregulated transcriptional response, with relative suppression of myeloid and monocyte signatures and enrichment of natural killer cell-associated genes. In addition, natural dengue cohorts from the same analysis further showed downregulation of innate immune and interferon signalling and higher cell-cycle activity, suggesting distinct innate immune transcriptomic profiles induced upon natural dengue infection versus vaccination (103).

### Neutralising antibody responses

In terms of NAb responses after the first dose of TAK-003, GMTs in the pivotal phase 3 trial (DEN-301) peaked one month after the first dose against all serotypes in baseline seropositive participants (GMTs in seropositive participants at Month 1: DENV-1: 2404 [placebo: 430], DENV-2: 6697 [placebo: 744], DENV-3: 2255 [placebo: 349], DENV-4: 1303 [placebo: 222]; GMTs in seronegative participants at Month 1: DENV-1: 118 [placebo: 5.8], DENV-2: 6277 [placebo: 6.6], DENV-3: 194 [placebo: 5.5], DENV-4: 111 [placebo: 5.4]; **Figure 1**) (21–25). In addition, in two studies which evaluated NAbs prior to Month 1 (DEN-313 and DEN-205), GMTs against all serotypes rose within 15 days of the first dose, irrespective of baseline serostatus, and peaked by Month 1 (76, 82).

Six studies reported seropositivity rates after the first dose (**Supplementary Table 2**). Tetravalent seropositivity was observed in 71.7%–95.2% of baseline seronegative participants in studies performed in both endemic and non-endemic regions, one month after receipt of the first dose, and 85.4%–99.1% in baseline seropositive participants (**Supplementary Table 2**).

### Non-neutralising antibody responses

Exploratory analysis from DEN-203 (using an earlier vaccine formulation) showed TAK-003 also induced total binding IgG, IgA, IgM, polyclonal IgG avidity, CFAs and anti-NS1 antibodies within the first 30 days post-vaccination in both adults and children from endemic regions and across baseline serostatus (67, 68, 70). Among these responses, IgM isotype responses peaked within 30 days, irrespective of baseline serostatus (68).

### T cell responses

Three studies evaluated T cell responses in the first month post-vaccination (DEN-205, DEN-210 and DEN-313) (123). Multifunctional cytokine (i.e. secretion of ≥2 cytokines of IFN-γ, tumour necrosis factor [TNF]-α, and interleukin [IL]-2) secreting T cell responses to both structural and NS proteins peaked early between Day 15 and Day 30 (Month 1) in dengue seropositive and flavivirus seronegative adults from a non-endemic setting (DEN-210), with IFN-γ ELISPOT T cell responder rates to any peptide pool spanning sequences of prME, NS1, NS3, and NS5 from all four serotypes of 100% at Month 1 post-vaccination (92). While not assessed as early as Day 15, IFN-γ ELISPOT T cell responses against all serotypes increased rapidly by Month 1 in 4–16 year-old children/adolescents from dengue endemic settings (DEN-313), with responder rates of 92% and 100% against any DENV peptide pool spanning non-structural proteins NS1, NS3 and NS5 at Month 1 in baseline seropositive and seronegative participants, respectively (82) (**Supplementary Figure 5**).

Collectively, although most of the studies were not specifically designed to evaluate immunogenicity after a single dose, these findings indicate that a single dose of TAK-003 activates coordinated innate, humoral, and cellular immunity across baseline serostatus and age in both dengue endemic and non-endemic settings.

## Immune responses following two doses of TAK-003

### Neutralising antibody responses

In total, 10 studies which evaluated NAb responses to TAK-003 using the final formulation/schedule were eligible for inclusion (**Table 1**). In the pivotal phase 3 study (DEN-301), receipt of a second dose had a slight impact on GMT titres, which was most pronounced in baseline seronegative participants. Prior to the second dose at Month 3, small decreases in GMTs against DENV-1, -3, and -4 were observed (seropositive DENV-1: 1945, DENV-3: 1563, DENV-4: 1002; seronegative DENV-1: 91, DENV-3: 94, DENV-4: 63), which then increased back to post-first dose levels by one month after the second dose (Month 4 seropositive DENV-1: 2115, DENV-3: 1761, DENV-4: 1129; seronegative DENV-1: 184, DENV-3: 228, DENV-4: 144). Decreases between doses were more pronounced against DENV-2 (Month 3 seropositive: 4826, seronegative: 1682) with little impact of the second dose (Month 4 seropositive: 4897, seronegative: 1730; **Figure 1**) (22). The relatively limited impact of the second dose on GMT titres was corroborated by data from the DEN-204 study, where the group receiving the 0 and 3 month schedule showed similar GMTs to the group who received one dose of vaccine, regardless of serostatus (74). In contrast, administration of the second dose at Year 1 led to greater boosting of non-DENV-2 titres in baseline seronegative participants compared to the second dose at 3 months (72). Although differing study designs make direct comparisons of GMTs across studies difficult, observed GMT responses to DENV-2 were generally higher than those for the other serotypes across the clinical development programme (**Figure 1**, **Supplementary Figures 2**, **3**) (25, 72, 82, 92, 95–99). GMT responses were also generally lower at all timepoints for baseline seronegative vs baseline seropositive participants.

In terms of seropositivity rates, the second dose increased tetravalent seropositivity in baseline seronegative participants. Across studies in endemic and non-endemic regions, 90.9%–99.6% of baseline seronegative participants achieved seropositivity against all four serotypes one month after completion of the two-dose series (Month 4), while rates in baseline seropositive participants (evaluated in endemic regions only) were already very high after receipt of the first dose (85.4%–99.1%) and remained high at all subsequent time points (76.2%–100%) (**Supplementary Table 2**). Observed seropositivity rates post-second dose by baseline serostatus were similar across the clinical studies and, while only representing a means of detection of measurable antibodies rather than any measure of protection from disease, informed the use of the three-month interval between two doses to maximise the potential for early protection, particularly for dengue-naive participants such as young children living in endemic regions.

Further, exploratory analysis using the anti-DENV-2 antibody depletion assay showed that TAK-003 induced both TS and CR NAbs to the non-backbone serotypes (DENV-1, DENV-3, and DENV-4), with percentage of non-DENV-2 TS NAbs at Month 4 ranging from 47.4% to 94.4% in 4–16 year-olds, and from 68.0 to 91.3% in 18–60 year-olds (**Supplementary Table 3**) (65).

### Non-neutralising antibody responses

Further exploratory assessments of TAK-003–induced humoral immunogenicity in the DEN-203 (earlier vaccine formulation), DEN-301, and DEN-304 studies showed that total binding IgG, IgA, and IgM, polyclonal IgG avidity, CFAs, and anti-NS1 antibodies slightly increased and remained stably detectable after the second dose in both baseline seropositive and seronegative participants (67, 68, 70, 104). Vaccination induced IgG, IgM, IgA and CFA responses were detected against all four serotypes up to a year post-vaccination, regardless of baseline serostatus (68), with similar titres by serotype and age detected in 4–16 year-olds and 18–60 year-olds, irrespective of baseline serostatus (104). In addition, TAK-003 induced IgG affinity-matured binding antibodies against all four serotypes, regardless of baseline serostatus and age (**Supplementary Table 3**) (103). These antibodies were shown to contributed to TAK-003-mediated virus neutralisation (70), as well as complement fixation and deposition (68).

Analysis of sera from children and adults (1.5 to 45 years) enrolled in the DEN-203 study showed that TAK-003 elicits anti-NS1 antibodies to DENV-2 NS1 of the vaccine backbone, which were cross-reactive against NS1 from the other DENV serotypes (67). Further evaluations as part of an immunobridging analysis comparing immunogenicity of TAK-003 in 4–16 year-olds in DEN-301 and 18–60 year-olds in DEN-304 demonstrated that the magnitude of NS1 IgG responses were comparable across age, with NS1 IgG concentrations at Month 4 of 246, 1053, 219 and 178 in children/adolescents and 299, 1300, 210, and 144 in adults, against DENV-1, DENV-2, DENV-3, and DENV-4, respectively (**Supplementary Table 3**) (104). This TAK-003 induced NS1-specific IgG has been also shown to block NS1-mediated endothelial hyperpermeabilisation *in vitro* (67).

### T cell responses

**Figure 2:**
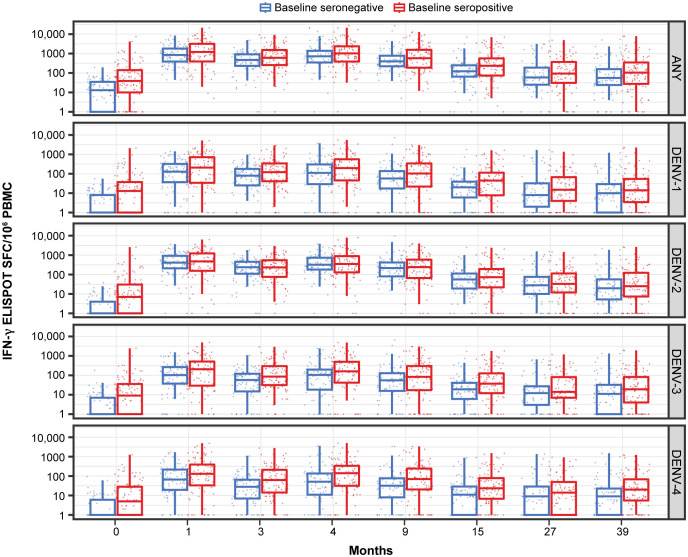
Median magnitude (error bars: interquartile range) of IFN-γ ELISPOT T cell responses to DENV peptide pools matching any or individual DENV serotypes in children and adolescents from first vaccination to Month 39, by baseline serostatus. ELISPOT enzyme-linked immunospot, IFN-γ interferon-gamma. Limit of detection: 5 SFC/10^6^ PBMC. The terms “seropositive” and “seronegative” refer to baseline dengue serostatus. Seropositivity was defined as a neutralising titre MNT ≥ 10 for at least one DENV serotype. Data are from DEN-313 performed in dengue-endemic regions (Panama and the Philippines) in baseline seropositive and seronegative children/adolescents (N = 200). Peptide pools evaluated in DEN-313: NS3 and 5 of DENV-1, -2, -3, and -4; NS1 of DENV-2. Reference: *Mandaric S*, et al. *NPJ Vaccines. 2024 Oct 17;9(1):192*.

In total, data on T cell responses following two doses of TAK-003 are available from three studies which evaluated the final formulation of TAK-003 (DEN-204, DEN-210, and DEN-313; **Table 1**), two of which directly evaluated IFN-γ ELISPOT T cell responder rates. In the long-term DEN-313 phase 2 study, performed in a population designed to emulate that of the phase 3 efficacy study (i.e. 4–16 year-olds living in dengue-endemic regions of Asia and Latin America), TAK-003 vaccination induced CD4+ and CD8+ T cell responses to all four DENV serotypes (overall IFN-γ ELISPOT responder rate one month after the second dose: 97.1% [95% CI 93.4–99.1%), which persisted through 3 years post-vaccination, regardless of the baseline serostatus (**Figure 2**) (82). The median IFN-γ ELISPOT magnitude to any peptide pool representing the four serotypes was higher for seropositive participants compared with seronegative participants (1086 vs 700, respectively). The highest responder rates and magnitudes of response were seen against DENV-2 and were lower but similar against the other three serotypes (**Supplementary Figure 4** and **5**). Similar findings were confirmed in the DEN-210 study in adults (18–60 years) from a dengue non-endemic region (92), with tetravalent T cell responder rates and magnitudes observed in flavivirus seronegative and baseline dengue seropositive participants maintained up to one year post-vaccination (**Supplementary Figure 4** and **5**).

Multifunctional cytokine-secreting (i.e. secretion of ≥2 cytokines of IFN-γ, TNF-α, and IL-2) CD4+ and CD8+ T cell responses were induced in both studies, as well as in the DEN-204 study, which characterised T cell responses in a subset of children aged 2 to 17 years. In all three studies, responses were consistent across baseline serostatus (75, 82, 92). Vaccine-elicited CD8+ T cell responses were of greater magnitude than CD4+ T cell responses, as expected for a live attenuated vaccine, with dominant phenotypes being IFN-γ+/TNF-α+ mostly targeting NS3 and NS5, regardless of the baseline serostatus and age group (75, 82, 92). Similarly, vaccination induced CD4+ T cells producing IFN-γ, TNF-α, and IL-2 cytokines to NS1, NS3 and NS5. Although NS proteins were the predominant targets of the vaccine-elicited T cells, multifunctional T cell responses targeting DENV structural proteins (DENV-1, -2, -3, and -4 prM and E and DENV-2 C) were also evaluated and observed in the DEN-210 study (92).

Taken together, two doses of TAK-003 induced functional humoral and cellular immune responses across age groups and endemicity settings, regardless of baseline serostatus.

## Long-term persistence and durability of immune response to vaccination

In dengue-endemic areas, DEN-301 evaluated antibody responses to the initial two-dose series up to 4.25 years (51 months) after receipt of the first dose of TAK-003 in 4–16 year-olds living in endemic areas. After peaking at Month 1 (after the first vaccine dose) or Month 4 (after the second dose), GMTs for all four DENV-serotypes plateaued and persisted through the observation period, up to 4.25 years (**Figure 1**) (21–25). Similar findings were observed in DEN-204, with persistence of immune responses in 2–17 year-olds over a 4 year period, from both the one- and two-dose schedules (72); and in DEN-313, where GMTs of NAbs were substantially elevated above baseline levels in 4–16 year-olds through 3 years post-vaccination, irrespective of serotype or baseline serostatus (**Supplementary Figures 2**, **3**) (82).

In dengue non-endemic regions, the DEN-303 study, which evaluated antibody responses in adolescents and adults (13–63 years), also showed antibody persistence up to 3.5 years (adults) or 5.75 years (adolescents) follow-up after the first dose of TAK-003 (94).

**Figure 3:**
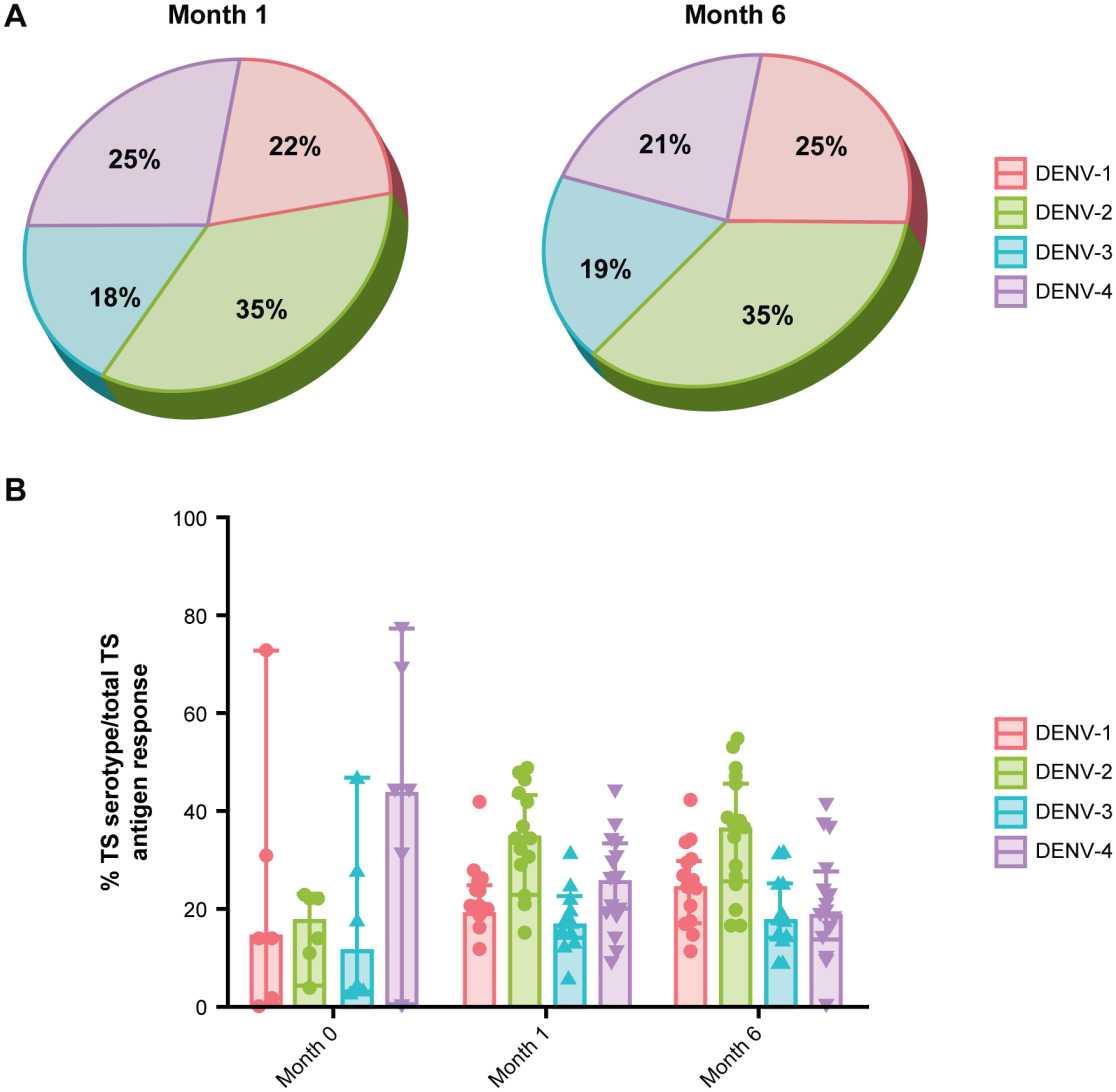
**(a)** Representative percentage of DENV type-specific memory B cell responses over the total type-specific response and **(b)** percentage of DENV type-specific responses over total type-specific antigen; in seronegative adults at Month 1 and Month 6 post-vaccination with TAK-003, by dengue serotype. TS, type specific. a) Proportion of DENV type-specific (TS) IgG-secreting memory B cells (MBCs) against the total TS response (100%) 1 month and 6 months post-single dose vaccination in representative seronegative adult participants from the DEN-205 study conducted in Singapore. The percentages of TS MBCs over each DENV serotype against the total TS response (100%) are shown in the pie charts. b) The percentage of DENV TS IgG secreting MBCs per serotype over total TS antigen for all participants are shown in the bar chart. Reference: *Michlmayr D*, et al. *J Infect Dis. 2021 Feb 3;223(2):247-257*.

In addition to NAbs, TAK-003 has also been shown to induce memory B-cells in exploratory analysis from DEN-205 study, both seropositive and seronegative participants developed DENV-specific TS and CR MBCs after vaccination. About two-thirds of post-vaccination responses were TS MBCs, persisting for at least 6 months regardless of the baseline serostatus. Unlike DENV infection, TAK-003 induced MBC responses against all four serotypes, regardless of prior dengue exposure (66) (**Figure 3**). MBCs reacted evenly to all four DENV serotypes (19–35% each), suggesting all vaccine components contribute to the immune response.

In terms of T cell responses, maintenance of IFN-γ ELISPOT responder rates against all four serotypes persisted up to 3.25 years post-first dose of TAK-003 in the DEN-313 study, irrespective of baseline serostatus (responder rate to any peptide pool at 3.25 years baseline seropositive: 74.2%, baseline seronegative: 82.4%; **Supplementary Figure 5**). In addition, multifunctional IFN-γ, TNF-α and IL-2 cytokine secreting CD4 and CD8 T cells also persisted through 3.25 years post-first dose, and were comparable between baseline seropositive and baseline seronegative participants (82). Initial data of participants included in the cell-mediated immunogenicity subset of the DEN-303 study also showed persistent T cell IFN-γ ELISPOT responses up to 3 years post-primary vaccination series (93).

Overall, the persistence of TAK-003-induced humoral and cellular immunity has been evaluated over multiple years in both endemic and non-endemic areas. Across studies, TAK-003-induced humoral and cellular immune responses persisted for up to 5.75 years post-vaccination, regardless of age, baseline serostatus, or region.

## Relationships between immunogenicity and protection

### Investigating correlate of protection against symptomatic dengue

As part of the exploratory analysis of data from the pivotal phase 3 DEN-301 study, GMTs from TAK-003 recipients who contracted VCD from one to 18 months after receipt of the two-dose series were compared with those of recipients who did not have VCD during that same time period (24). While no clear threshold could be identified from the data, participants who experienced VCD generally had lower NAb titres than those who did not have VCD, although this was largely driven by data from baseline seropositive participants. Further analysis assessing the risk of VCD based on low, medium, and high terciles of NAb titre levels pooled across the 4 serotypes, also showed a trend towards lower risk of VCD with higher antibody titres. (**Supplementary Table 4**) (24).

Further, exploratory evaluation of T cell and NAb immune responses in a small number of TAK-003 recipients who developed VCD in the DEN-313 study showed that nine of 12 VCD cases had lower IFN-γ ELISPOT T cell responses and seven had lower NAb GMTs than the median of non-VCD participants (82).

While the exact mechanism of action of TAK-003 on each of these individual components of the immune system remains to be further elucidated, exploratory analyses suggested lower immune responses among participants who developed VCD than among those who did not.

### Investigating correlates of protection against severe dengue

Despite some decline in TAK-003 overall efficacy against symptomatic dengue from the first year to second year post-vaccination in DEN-301, TAK-003 efficacy against hospitalised dengue remained robust, in both baseline seropositive (vaccine efficacy: 85.9%) and seronegative (vaccine efficacy: 78.3%) recipients up to 4.5 years post-vaccination; with no increase in severe dengue incidence in TAK-003 recipients compared with placebo (25). In addition, TAK-003-induced NS1-specific IgG has been shown to block virus-mediated vasculopathy *in vitro* across the serotypes, irrespective of baseline serostatus (67), suggesting a potential mechanism that may contribute to protection against severe dengue (56). TAK-003 vaccination induced tetravalent CFAs regardless of serostatus and age (68, 104). In natural infection, the magnitude of CFAs has been associated with protection from severe dengue (105, 106), although whether this association translates into a robust correlate of protection in vaccine recipients remains uncertain. TAK-003 also induced multifunctional T cells expressing the proinflammatory cytokines IFN-γ and TNF-α, which have been associated with reduced susceptibility to severe dengue in natural infection (51). Collectively, these vaccine-elicited immune responses may contribute to protection against hospitalisation and possibly severe dengue; however, the available evidence remains indirect.

## Immune responses to vaccination by age

As part of the clinical development of TAK-003, an immunobridging analysis was performed which compared NAb responses of 702 baseline seronegative children and adolescents (aged 4–16 years) enrolled in DEN-301 with those of 379 adults (18–60 years) in the DEN-304 study, which was performed in areas of the US considered non-endemic for dengue (104). Comparable GMTs against individual dengue serotypes at Month 4 (one month after the second dose) and Month 9 (6 months after the second dose) were observed across the two age groups (**Supplementary Figure 6**), indicating similar immune responses to vaccination in children and adults, and therefore suggesting that a similar vaccine efficacy could be expected across the age groups. Additional exploratory analysis further characterising the humoral response showed comparable magnitudes of IgG binding, NS1 and complement-fixing antibody titres across the age groups, with TAK-003 eliciting both type-specific and cross-reactive antibodies in both children and adults (**Supplementary Table 3**). Assessment of IgG avidity, a measure of the binding strength of induced antibodies, showed that the tetravalent binding antibodies elicited by TAK-003 were affinity-matured in both the paediatric and adult groups (104).

Further subgroup analysis specifically evaluating NAb responses by age group was also performed in the DEN-301 and DEN-204 studies. In DEN-301, seropositivity rates post-vaccination were similar across age groups (4–5 years, 6–11 years, and 12–16 years), irrespective of baseline serostatus. While NAb GMTs were also similar across age groups in baseline seronegative participants, there was some variation in baseline seropositives, with highest GMTs in 12–16 year-olds and lowest GMTs in 4–5 year-olds, likely due to the accumulation of DENV exposures with age (77). In the group of children who received the final formulation of TAK-003 in the DEN-204 study, trends in GMTs over time were similar across age groups, including in children aged 2–5 years (72).

In terms of T cell responses, no clear differences in IFN-γ ELISPOT responses rates were seen in subgroup analysis of data from the DEN-313 study, with similar rates of positive IFN-γ ELISPOT responses across age groups (Month 4, 4–5 years responder rate baseline seropositive: 96.7% seronegative: 100%; 6–11 years seropositive: 95.4%, seronegative: 97.4%; 12–16 years seropositive:100%, seronegative: 100%) (82). Additional cross-study exploratory analysis comparing T cell responses in the baseline seronegative participants in the DEN-313 (children 4–16 years) and DEN-210 studies (adults 18–60 years) also showed comparable TAK-003-elicited T-cell responses to individual dengue serotypes in children and adults, though the small number of adult participants should be taken into consideration when interpreting these findings (107).

## Impacts of other vaccines

### Coadministration

Three studies have evaluated the impacts of immunogenicity when TAK-003 was coadministered with a range of representative vaccines including a live vaccine (yellow fever) (96), inactivated vaccine (hepatitis A) (98), and recombinant multivalent vaccine (HPV) (81). All of these studies showed that coadministration of TAK-003 with other vaccines was non-inferior to administration of the established vaccine alone, with limited or no impact on TAK-003-induced neutralising antibody titres.

#### Yellow fever

The DEN-305 study evaluated the non-inferiority (defined as upper bound of 95% CI for between-group differences in seroprotection rate <5% at Month 1) of immunogenicity to yellow fever vaccine (YF-17D) when administered concomitantly with TAK-003 versus alone in a population of 900 adults aged 18–60 years living in areas of the US non-endemic for dengue. In total, 99.5% (95% CI 97.4–100%) of participants who received YF-17D in combination with TAK-003 achieved yellow fever antibody titres considered seroprotective one month after vaccination compared with 99.1% (96.9–99.9%) who received YF-17D alone. GMTs against individual DENV serotypes were non-inferior (defined as upper bound of 95% CI for GMT ratio <2 at Month 1) when TAK-003 was administered alone or with the yellow fever vaccine (YF-17D), except against DENV-1 where titres were marginally lower following concomitant administration (GMT ratio 1.6 [95% CI 1.19–2.22]) (96). However, the immune response to DENV-1 was comparable to that seen in the clinical development program, including the pivotal efficacy trial among participants who were baseline seronegative for DENV (21).

#### Hepatitis A

In the DEN-314 study, which evaluated the immunogenicity and safety of coadministration of TAK-003 with inactivated hepatitis A vaccine in 900 adults aged 18–60 years in the UK, seroprotection rates following vaccination against hepatitis A were similar with concomitant administration of TAK-003 or alone. The primary objective, non-inferior (upper bound of the 95% CI for difference in seroprotection rate <10%) seroprotection rate in the coadministration group versus hepatitis A vaccine alone was met, with seroprotection rates against hepatitis A of 97–98% in both groups, resulting in a difference of −1.68% (95% CI −8.91, 4.28%). Tetravalent seropositivity against dengue was 91–97%, indicating no clear impacts of coadministration on the immunogenicity of either vaccine (98).

#### HPV

Similarly, in the DEN-308 study evaluating co-administration of TAK-003 with the 9-valent human papillomavirus (HPV) vaccine in 614 children and adolescents aged 9–14 years in a dengue-endemic setting (Thailand), post-vaccination HPV total immunoglobulin G (IgG) levels were similar with co-administration or HPV vaccine alone. Non-inferiority of responses was concluded as the upper bound of the 95% CI for total HPV IgG ratio between coadministered and single-vaccine groups was <1.5 for all 9 HPV types. Overall, 99.6% of participants achieved tetravalent dengue seropositivity across groups, with GMTs broadly comparable with those observed from other studies, taking into account differences in study designs and assay variabilities (81). While neither this study nor the study evaluating coadministration of TAK-003 with hepatitis A vaccine included a TAK-003 only arm, dengue-specific antibody titres in both studies were consistent with those observed in other studies in the TAK-003 clinical development program.

### Previous receipt of other flavivirus vaccines

In a subgroup analysis of data from the DEN-301 study, no clinically-relevant impacts of prior yellow fever or Japanese encephalitis vaccination were seen on TAK-003 efficacy in the ~9000 children and adolescents who had been previously vaccinated (yellow fever ~4200, Japanese encephalitis ~4800). Irrespective of baseline serostatus, the kinetics of GMTs against each of the dengue serotypes were similar in participants with or without prior yellow fever or Japanese encephalitis vaccination (79).

## Impact of a booster dose

Further evaluations have now been performed assessing the effects of a booster dose given 3 to 5 years post-primary vaccination series (108–110). As part of the pivotal efficacy study (DEN-301), participants aged 4–11 years at the start of the study were offered a booster dose of TAK-003 or placebo 4 to 4.5 years after receipt of the primary series. In total, 8977 participants were randomised, of whom 5991 received TAK-003. The booster dose elevated GMTs against all four serotypes one month after vaccination, which then reduced with time but remained above pre-booster levels up to 2 years and one month (25 months) post-booster (108). In DEN-303, consenting adolescents and adults who had previously been enrolled in DEN-304 or DEN-315 were randomised to receive one booster dose of TAK-003 or placebo, 3 years (DEN-304) or 5.25 (DEN-315) years after their first dose of TAK-003 (93). Overall, 233 participants received the booster vaccination, of whom 115 received TAK-003. Across both studies, the booster dose resulted in increased antibody titres compared with pre-booster levels, followed by some decline over time. In baseline seronegative participants, the booster dose elicited higher peak antibody titres against non-DENV-2 serotypes in comparison to that achieved after the primary series, regardless of the timing of booster dose. These titres were comparable to the peak antibody titres achieved after using a vaccination schedule with doses at 0 and 12 months (72). In baseline seropositive participants, the booster dose did not have a significant impact on the peak antibody titres compared with those achieved after the primary series. Despite the observed increased antibody titres after receipt of a booster dose, relatively modest impacts were seen on vaccine efficacy against symptomatic disease, and very limited impacts were seen against hospitalised dengue (93, 109, 110).

## Variability of immune responses

Variability in NAb GMTs following vaccination was observed during the clinical program, attributable to both differences in individual immunological responses and inherent variability of the validated MNT assay (111). While some participants developed strong antibody responses, others showed only modest increases, resulting in heterogeneity and wide confidence intervals for GMTs. This variability was further influenced by baseline serostatus – baseline seropositive recipients generally had higher and sometimes more variable GMTs, reflecting differences in the timing and number of prior DENV exposures. Similarly, a large variation was observed in the magnitude of T cell responses during the clinical development program, despite overall high levels of T cell responder rates (75, 82, 92). Despite this variability, the pivotal trial (DEN-301) showed efficacy across dengue-endemic regions in different epidemiological settings across Asia Pacific and Latin America regions, highlighting the complex interplay of immune factors in mediating protection against dengue.

## Discussion

Given the inherent complexity of dengue immunity, TAK-003 has been extensively characterised for a comprehensive array of functional metrics of humoral and cellular immunity throughout its clinical development. This body of work was designed not only to support regulatory evaluation of the vaccine, but also to generate a broader understanding of how a live attenuated tetravalent dengue vaccine shapes immune responses across baseline serostatus, age, and geography. In addition to the standard immunogenicity assessments needed to meet regulatory requirements, TAK-003 has also been further assessed to provide additional insight into its potential mechanism of action in relation to dengue immunology, and how this likely relates to immune responses following natural infections. Accordingly, this review synthesises both the robust trial findings and exploratory analyses, while distinguishing between established observations and hypotheses that remain under investigation. Across the clinical development program, TAK-003 has shown persistent humoral and T cell induced-immunogenicity against all four dengue serotypes, in both dengue-naive and pre-exposed participants from dengue endemic regions of Asia and Latin America, and non-endemic regions. The most robust findings come from the pivotal phase 3 and long-term follow-up studies, which show that TAK-003 induces neutralising antibodies and T-cells to all four serotypes, with generally higher responses against DENV-2 and comparable against other DENV serotypes, and that these responses persist for several years after vaccination. Although the magnitude of NAb titres and IFN-γ ELISPOT responses are not directly comparable across studies, TAK-003 induced NAb and T cell responses to all four DENV serotypes regardless of baseline serostatus, age and region. In additional exploratory analyses from smaller immunogenicity subsets, TAK-003 also elicited high avidity anti-DENV binding antibodies; tetravalent complement fixing antibodies; functional antibodies to DENV-2 NS1 that cross-react with NS1 of DENV-1, DENV-3, and DENV-4 and memory B cells specific to all four serotypes.

Collectively, TAK-003’s extensive immunogenicity analysis provides insight into an immunological basis for its observed efficacy and safety profiles. However, the available evidence does not yet establish a validated correlate of protection. In terms of early responses to vaccination after the first dose, onset of efficacy was observed in the first two weeks of the DEN-301 phase 3 clinical trial, with 82.1% efficacy against infection observed in the 3 months between the first and second doses (112). This response is corroborated by real-world data, with protection against symptomatic dengue observed within 14 days after the first dose (vaccine efficacy 67.4% at 14–27 days) during a large outbreak in 2024 in Brazil (26). Immunogenicity findings following the initial dose of TAK-003 also support this observed early protection, with upregulation of the type I IFN pathway and early induction of T cell responses, antibody and NS1 responses within 2–4 weeks post-vaccination, although these analyses were generally exploratory in nature. Recent research into gene expression in DENV infections and TAK-003 vaccination has also shown that innate immune gene expression profiles are altered after primary DENV infection but not after tetravalent vaccination with TAK-003 (103). Natural infection was shown to alter the expression of pro-inflammatory innate immune markers, including those related to dengue pathogenesis, while TAK-003 vaccination did not show such patterns. However, further evaluations are needed to clarify how immune imprinting and prior exposure shape vaccine-induced immune responses relative to natural infection and how those responses translate to protection from disease.

While further research is needed before any correlate of protection can be established for dengue, vaccine-induced effects on different arms of the immune system can provide important insights into identifying mediators associated with disease protection. For example, a trend towards higher NAb titres and lower risk of dengue was noted in the DEN-301 pivotal study. Further, DENV-specific T cell responses have also been possibly linked to protection against dengue and reduced risk of severe dengue in natural infection (41) and it is possible that DENV-specific T cells play an important role in preventing severe disease when NAb titres are insufficient to block breakthrough infections. In line with this, a recent real-world observational study comparing immune profiles in TAK-003-vaccinated travellers, with or without prior natural dengue exposure, versus non-vaccinated travellers experiencing acute natural dengue infection found that TAK-003 recipients had higher DENV-specific T-cell responses than the non-vaccinated acute-infection group at 3 months after the first dose (113). Other elements of the immune response including total binding IgG, IgA, and IgM, polyclonal IgG avidity, CFAs, and anti-NS1 were also noted to be induced by TAK-003, and remained stable over time, regardless of baseline serostatus. These responses are notable because they extend beyond simple neutralisation and may contribute to broader antiviral function; however, their clinical relevance in vaccine recipients remains uncertain. These humoral functions are now considered important contributors to protection against symptomatic dengue and severe disease in natural infections, including high-avidity opsonization of virus, inhibition of NS1-driven vasculopathy, and engagement of innate immune mechanisms that promote complement activation and effector-cell–mediated clearance of infected cells. Whether these same functions make a measurable contribution to protection after vaccination has not yet been established.

When assessing immunological memory, TAK-003 induced memory B cells to all four DENV serotypes, consistent with the establishment of tetravalent B cell immunological memory. This is an important finding because it suggests the vaccine can generate recall-capable responses across the serotypes, but the durability and clinical consequences of this memory remain to be confirmed in real-world settings. Additional exploratory evidence also showed that TAK-003 elicited IgA and IgM responses suggest induction of effective DENV memory, in line with data from natural DENV exposures (47). Taken together with the effects on various immune mediators described above, these findings highlight the importance of characterising the role of multiple elements of the dengue immune response in the context of tetravalent vaccination, in order to elucidate the immunological mechanism(s) leading to the protection from infection and/or disease demonstrated in phase 3 clinical trials. At the same time, the current evidence base remains insufficient to determine which of these responses are most predictive of protection, or whether some are simply biomarkers of exposure.

Live vaccines such as TAK-003 can mimic elements of natural infection, leading to a strong and sustained immune response across all dengue serotypes, with a trend suggesting a favourable effect of TAK-003 on clinical manifestations of breakthrough cases (23, 78). While breakthrough cases did occur in TAK-003 recipients, in the ~4.75 years follow-up of the DEN-301 study, TAK-003 recipients were substantially less likely to experience sequential symptomatic breakthrough cases than placebo recipients (relative risk 0.19, 95% CI: 0.07–0.54) suggesting a potential benefit of TAK-003 beyond prevention of an initial breakthrough case (78). TAK-003 has also been shown to provide benefits against asymptomatic infection with efficacy varying from 27%–51% at four to nine months after the first dose, depending on the model used (114). Although the vaccine may not provide sterilising immunity, it appears to elicit immune responses which reduce the incidence and severity of symptoms, although the exact mechanisms of action remain to be elucidated. Moreover, continued dengue transmission in endemic areas may provide natural boosting to vaccine recipients, thereby helping to further prevent breakthrough infections and cases of severe dengue.

While viremia generally occurs during natural DENV infection (1) and could therefore be considered as necessary for an effective response to vaccination, TAK-003 vaccine viremia (e.g. detectable RNAemia in peripheral blood) was generally only detected for DENV-2 (76), but immunogenicity, including TS responses, was induced across all 4 DENV serotypes (65, 66). Interestingly, efficacy was seen against DENV-1 in baseline seronegative participants, despite no previous detectable viremia (21, 25). This suggests that a lack of detectable viremia in peripheral blood is not associated with a failure to mount an effective immune response. The induction of tetravalent immunity following vaccination likely occurs through local viral replication within secondary lymphoid organs, which play a crucial role in the development of adaptive immunity, although the details of how TAK-003 mediates this process are yet to be elucidated.

Cross-reactive immunity has been observed among flaviviruses. Cross-reactive antibodies and multitypic T cells can contribute to cross-protection when they exhibit sufficiently strong effector functions (115). However, cross-reactivity may also be non-protective or even pathogenic, particularly in dengue (116). TAK-003, in addition to induction of type-specific humoral immunity to all four dengue serotypes, also induced cross-reactive humoral and cellular immunity. These responses did not appear to result in pathogenic effects, as no increase in severe dengue has been observed among TAK-003 vaccinees during the long-term follow-up in clinical trials (25, 109, 110), as is continuing to be monitored as part of post-marketing pharmacovigilance.

While not a key objective of TAK-003 immunogenicity evaluations, exploratory analysis failed to identify thresholds for correlate of protection. Within the immune response, there is an interplay between multiple arms operating synergistically to clear viral infections and/or prevent development of severe disease; thus, immune responses that confer protection against infection might differ from those that protect against severe disease (117). While NAbs are used as a key measure of vaccine immunogenicity, other immune arms are also important in neutralising dengue and preventing disease progression. For example, while CYD-TDV, which is based on a yellow fever backbone, showed robust anti-DENV NAb responses (118), one hypothesis as to why a heightened severity of dengue infection was observed in seronegative participants following CYD-TDV vaccination was related to the lack of dengue-specific NS proteins in the CYD-TDV vaccine backbone and subsequent lack of induction of robust anti-DENV T cell immunity (119, 120). In contrast, the inclusion of DENV NS proteins in TAK-003 resulted in the induction of T cell responses across all four serotypes, with further analysis required to establish whether these T cell responses could play a role in establishing a correlate of protection against symptomatic and/or severe dengue. In addition, the impact of inter-subject variability of vaccine-induced immune responses on the correlate(s) of protection remains to be elucidated. These findings highlight the continued need for large-scale efficacy studies and a comprehensive evaluation of the immune response to better understand the protection conferred by dengue vaccines. They also suggest that a correlate of protection may be complex, involving multiple components of the immune system, and dependent on numerous factors including different thresholds per infecting serotype, past exposure(s) and time since exposure, and disease manifestations. Importantly, this complexity means that the present review should be interpreted as a synthesis of available evidence rather than as a definitive mechanistic model.

Despite comprehensive assessments of immunogenicity throughout the clinical development programme, there remain some unanswered questions and uncertainties in TAK-003-induced protection from dengue. Firstly, in the pivotal DEN-301 study, TAK-003 demonstrated efficacy in participants with prior exposure to all DENV serotypes, but efficacy in dengue-naive participants could be demonstrated only against DENV-1 and DENV-2, with no suggested efficacy against DENV-3 and too few cases of DENV-4 for evaluation (25). While it should be noted that the serotype- and serostatus-stratified analyses were exploratory, and the lower incidence of DENV-3 and DENV-4 cases among dengue-naive participants during the study limited robust conclusions for these serotypes in that population, it was interesting to observe that efficacy for DENV-1 but not DENV-3 in the dengue-seronegative population, despite similar NAbs responses (25). This disparity has also been observed for other dengue vaccines, with efficacy results for the investigational live-attenuated tetravalent Butantan-dengue vaccine, which includes full-genome DENV-1, DENV-3, DENV-4, and a DENV-2/DENV-4 chimeric backbone, revealing a similar trend (120–122). Further immunogenicity evaluations of TAK-003 beyond NAbs alone also showed no clear differences in any of the immune components which could directly account for this variable efficacy. Further to these findings after the primary vaccine series, during the 2.1 year (25-month) follow up after a booster dose baseline seronegative participants demonstrated efficacy against DENV-4 (78.2% [29.4% to 93.3%]) but remained inconclusive against DENV-3 (37.9% [−85.0% to 79.1%]) (108). The performance of DENV-3 and-4 in baseline seronegative recipients is under further evaluation in an ongoing large-scale post-authorisation effectiveness study (NCT06843226). Additional studies also ongoing will assess immunogenicity and safety in different populations, including in children below 4 years of age [NCT06665035], adults above 60 years of age including comorbidities [NCT06579755], and across different geographies [NCT06741683] to gain further insight into any difference in immunogenicity across age groups or locations. Another consideration which should be taken into account when reviewing the immunogenicity evidence is differences in serotype circulation patterns and forces of infection across endemic areas. While the pivotal phase 3 trial was designed to incorporate this diversity by including a variety of endemic countries across Latin America and Asia, DENV serotype circulation varies over time and may also have contributed to the low incidence of cases of particular serotypes (e.g. DENV-4) in the trial. Finally, the lack of an established correlate of protection remains a major challenge in dengue vaccine development. While the exploratory studies performed as part of TAK-003 clinical development have provided further insight into the vaccine’s effects on immune responses, the mechanism(s) of action in prevention of symptomatic and severe disease remains to be fully elucidated. Future work integrating efficacy, real-world effectiveness, and systems-level immune profiling will be needed to determine which signals are genuinely predictive of protection.

This review has several limitations. As a narrative synthesis, it may be subject to some degree of interpretive bias despite efforts to remain comprehensive and balanced. In addition, some immunogenicity findings come from small exploratory subanalyses. Finally, although TAK-003 has been extensively evaluated immunologically, no validated correlate of protection exists for dengue, so mechanistic inferences remain hypothesis-generating, and the lack of established correlates of protection for dengue limits interpretation of immunogenicity in relation to durability and long-term efficacy.

In conclusion, the clinical development programme of TAK-003 has evaluated an array of humoral and cellular mediators of immune responses to dengue, with immunogenicity evidence providing support to the observed long-term efficacy and safety profiles of the vaccine. While a number of unmet questions remain, including establishment of a correlate of protection for dengue and how immunogenicity to individual serotypes relates to efficacy, the evidence to date suggest that TAK-003-driven immunity supports the sustained long-term efficacy and safety profile of TAK-003 and its important role in a comprehensive dengue control program to reduce the global health burden of this disease.
