# Supplementary material for: Mapping immune responses to TAK-003 dengue vaccine: immunogenicity across the clinical development program
Source: Front Immunol. 2026 Jul 22;17:1851238. doi: 10.3389/fimmu.2026.1851238 (PMC13438160; doi:10.3389/fimmu.2026.1851238)
Supplement: Supplementary file 1 [file SupplementaryFile1.docx]

**Supplementary Materials**

**Table of Contents**

[Supplementary Figure 1. TAK-003 live-attenuated tetravalent dengue vaccine design	2](#_Toc233815191)

[Supplementary Figure 2. GMTs of neutralising antibody responses across studies DEN-313 and DEN-204 performed in children and adolescents in endemic regions, by dengue serotypes and baseline serostatus	3](#_Toc233815192)

[Supplementary Figure 3. GMTs of neutralising antibody responses across studies performed in adults and adolescents in non-endemic regions (baseline seronegative participants), by dengue serotype	4](#_Toc233815193)

[Supplementary Figure 4. Magnitude of IFN-γ ELISPOT T cell responses to DENV peptide pools matching any or individual DENV serotypes by baseline serostatus in adults from non-endemic regions, by dengue serotypes and baseline serostatus	5](#_Toc233815194)

[Supplementary Figure 5. IFN-γ ELISPOT T cell responder rates to any DENV peptide pool by baseline serostatus in (a) children and adolescents from endemic regions and (b) adults from non-endemic regions by dengue serotypes and baseline serostatus	6](#_Toc233815195)

[Supplementary Figure 6. Immunobridging analysis: comparison of GMTs across age groups at Months 4 and 9 between seronegative children and adults (9)	7](#_Toc233815196)

[Supplementary Table 1. Baseline characteristics of participants enrolled in TAK-003 studies using final formulation and dosing schedule contributing to immunogenicity evaluation in (a) endemic regions and (b) non-endemic regions	8](#_Toc233815197)

[Supplementary Table 2. Percentage of participants with tetravalent seropositivity (95% CI) for (a) studies performed in children and adolescents from endemic regions by baseline serostatus and (b) studies performed in adults from non-endemic regions (all baseline seronegative participants)	10](#_Toc233815198)

[Supplementary Table 3. Exploratory immunology assessments in children and adolescents in endemic regions and adults in non-endemic regions (baseline seronegative participants), by dengue serotype (9)	11](#_Toc233815199)

[Supplementary Table 4. Hazard ratios (95% CI) of correlate of risk of VCD analysis between 1 month after second dose and until 18 months after the second dose (approximately Month 21 after the first dose; per protocol set – correlate of protection subset data) and by baseline serostatus based on neutralising antibody titres one month after the second dose (Month 4) (23)	12](#_Toc233815200)

## Supplementary Figure 1. TAK-003 live-attenuated tetravalent dengue vaccine design

C, capsid; E, envelope; NS, non-structural protein; prM, pre-membrane; TDV-1/2/3/4, dengue serotype 1/2/3/4 strain.

5’-NCR-57: C-to-T mutation; NS1-53: Glycine-to-Aspartic acid mutation; NS3-250: Glutamic acid-to-Valine mutation.

Reproduced from Patel et al. Clinical safety experience of TAK-003 for dengue fever: a new tetravalent live attenuated vaccine candidate. *Clin infect Dis*. 2022 [May 26];ciac418. doi :10 .1093/cid/ciac418; © the Author(s) 2022. Originally published by Oxford University Press on behalf of the Infectious Diseases Society of America.

## Supplementary Figure 2. GMTs of neutralising antibody responses across studies DEN-313 and DEN-204 performed in children and adolescents in endemic regions, by dengue serotypes and baseline serostatus

GMT, geometric mean titre. Limit of detection: MNT=10. The terms “seropositive” and “seronegative” refer to baseline dengue serostatus. Seropositivity was defined as a neutralising titre MNT ≥ 10 for at least one DENV serotype. DEN-313 was performed in children aged 4–16 years in Panama and the Philippines (N=195). DEN-204 was performed in children aged 2–17 years in the Dominican Republic, Panama, and the Philippines (N=83) (1, 2).

## Supplementary Figure 3. GMTs of neutralising antibody responses across studies performed in adults and adolescents in non-endemic regions (baseline seronegative participants), by dengue serotype

GMT, geometric mean titre. . Limit of detection: MNT=10. The terms “seropositive” and “seronegative” refer to baseline dengue serostatus. Seropositivity was defined as a neutralising titre MNT ≥ 10 for at least one DENV serotype. Non-endemic studies were performed in adults aged 18–60 years in the UK (DEN-314, N=63) and the USA (DEN-304, N=447; DEN-307, N=139; DEN-305, N= 208; DEN-210, N=21), and in adolescents aged 12–17 years in Mexico City (DEN-315, N=271) (3-8).

## Supplementary Figure 4. Magnitude of IFN-γ ELISPOT T cell responses to DENV peptide pools matching any or individual DENV serotypes by baseline serostatus in adults from non-endemic regions, by dengue serotypes and baseline serostatus

ELISPOT enzyme-linked immunospot, IFN-γ interferon-gamma. Limit of detection: 5 spot-forming cells/10^6^ peripheral blood mononuclear cells

Data from DEN-210 performed in non-endemic regions of the USA in baseline seropositive and seronegative adults (N=30) (3). Peptide pools evaluated in DEN-210: prME, NS1, 3, and 5 of DENV-1, -2, -3, and -4; NS2, 4, and C protein of DENV-2.

## Supplementary Figure 5. IFN-γ ELISPOT T cell responder rates to any DENV peptide pool by baseline serostatus in (a) children and adolescents from endemic regions and (b) adults from non-endemic regions by dengue serotypes and baseline serostatus

ELISPOT, enzyme-linked immunospot; IFN-γ, interferon-gamma.

Endemic regions (Panama and the Philippines): data from DEN-313 in baseline seropositive and seronegative children/adolescents (N=200) (1). Non-endemic regions (USA): data from DEN-210 in baseline seropositive and seronegative adults (N=30)(3). A positive T cell response was defined as IFN-γ ELISPOT response >3 times higher than baseline and ≥5 spot forming cells [SFCs]/10^6^ peripheral blood mononuclear cells [PBMCs]). Limit of detection: 5 SFC/10^6^ PBMC. The terms “seropositive” and “seronegative” refer to baseline dengue serostatus. Seropositivity was defined as a neutralising titre MNT ≥ 10 for at least one DENV serotype. Peptide pools evaluated in DEN-313: NS3 and 5 of DENV-1, -2, -3, and -4; NS1 of DENV-2. Peptide pools evaluated in DEN-210: prME, NS1, 3, and 5 of DENV-1, -2, -3, and -4; NS2, 4, and C protein of DENV-2.

## Supplementary Figure 6. Immunobridging analysis: comparison of GMTs across age groups at Months 4 and 9 between seronegative children and adults (9)

GMT, geometric mean titre. . Limit of detection: MNT=10. The term “seronegative” refer to baseline dengue serostatus. Seropositivity was defined as a neutralising titre MNT ≥ 10 for at least one DENV serotype. DEN-301 was performed in dengue-endemic regions of Asia and Latin America (Brazil, Colombia, Dominican Republic, Nicaragua, Panama, Philippines, Sri Lanka, Thailand). DEN-304 was performed in regions of the USA considered non-endemic for dengue.

## Supplementary Table 1. Baseline characteristics of participants enrolled in TAK-003 studies using final formulation and dosing schedule contributing to immunogenicity evaluation in (a) endemic regions and (b) non-endemic regions

(a) Endemic Regions

| **Parameter** | **DEN-301 N = 2518** | **DEN-313 N = 195** | **DEN-204 N = 83** |
| --- | --- | --- | --- |
| Age (years) |  |  |  |
| Mean (SD) | 9.6 (3.36) | 6.6 (2.29) | 8.4 (4.19) |
| Range (min, max) | (4, 16) | (4, 15) | (2, 17) |
| Gender, n (%) |  |  |  |
| Male | 1275 (50.6) | 96 (49.2) | 36 (43.4) |
| Female | 1243 (49.4) | 99 (50.8) | 47 (56.6) |
| Race, n (%) |  |  |  |
| American Indian/Alaska Native | 949 (37.7) | 73 (37.4) | 38 (45.8) |
| Asian | 1173 (46.6) | 98 (50.3) | 16 (19.3) |
| Black or African American | 272 (10.8) | 8 (4.1) | 29 (34.9) |
| Native Hawaiian/ Other Pacific Islander | 0 | 0 | 0 |
| White | 55 (2.2) | 9 (4.6) | 0 |
| Multiracial | 69 (2.7) | 7 (3.6) | 0 |
| Body mass index (kg/m^2^) |  |  |  |
| Number of evaluable subjects | 2515 | 195 | 83 |
| Mean (SD) | 17.77 (3.890) | 16.20  (3.129) | 17.88  (3.948) |
| Median (min, max) | 16.80 (8.9, 48.5) | 15.40 (9.5, 32.1) | 16.81 (13.1, 31.6) |
| Baseline dengue serostatus |  |  |  |
| Positive, n (%) | 1816 (72.1) | 109 (55.9) | 41 (49.4) |
| Negative, n (%) | 702 (27.9) | 86 (44.1) | 42 (50.6) |

(b) Non-endemic regions

| **Parameter** | **DEN-304^(b)^**  **N = 447** | **DEN-307 N = 139** | **DEN-315 N = 271** | **DEN-305^(a)^ N = 208** | **DEN-314^(a)^ N = 63** | **DEN-210^(c)^ N = 21** |
| --- | --- | --- | --- | --- | --- | --- |
| Age (years) |  |  |  |  |  |  |
| Mean (SD) | 41.0 (12.02) | 41.1 (11.69) | 14.3 (1.67) | 40.5 (12.64) | 35.9 (11.35) | 33.6 (10.15) |
| Range (min, max) | (18, 60) | (18, 60) | (12, 17) | (18, 60) | (18, 59) | (22, 58) |
| Gender, n (%) |  |  |  |  |  |  |
| Male | 211 (47.2) | 61 (43.9) | 115 (42.4) | 82 (39.4) | 37 (58.7) | 11 (52.4) |
| Female | 236 (52.8) | 78 (56.1) | 156 (57.6) | 126 (60.6) | 26 (41.3) | 10 (47.6) |
| Race, n (%) |  |  |  |  |  |  |
| American Indian/Alaska Native | 2 (0.4) | 0 | 139 (51.3) | 0 | 0 | 0 |
| Asian | 4 (0.9) | 4 (2.9) | 0 | 3 (1.4) | 2 (3.2) | 2 (9.5) |
| Black or African American | 85 (19.0) | 29 (20.9) | 0 | 73 (35.1) | 0 | 1 (4.8) |
| Native Hawaiian/ Other Pacific Islander | 1 (0.2) | 1 (0.7) | 0 | 0 | 0 | 1 (4.8) |
| White | 347 (77.6) | 102 (73.4) | 0 | 129 (62.0) | 60 (95.2) | 17 (81.0) |
| Multiracial/other | 8 (1.8) | 1 (0.7) | 132 (48.7) | 0 | 1 (1.6) | 0 |
| Body mass index (kg/m^2^) |  |  |  |  |  |  |
| Number of evaluable subjects | 447 | 139 | 271 | 208 | 63 | 21 |
| Mean (SD) | 27.82  (4.336) | 27.50  (4.19) | 22.27  (4.211) | 27.16  (4.415) | 26.60  (4.435) | 24.74 (2.967) |
| Median (min, max) | 27.90 (17.7, 35.1) | 28.0  (19.0, 35.0) | 21.33 (14.3, 34.8) | 27.28 (18.2, 35.0) | 26.38 (18.0, 34.6) | 24.80 (19.4, 32.0) |
| Baseline dengue serostatus |  |  |  |  |  |  |
| Positive, n (%) | 68 (15.2) | 0 | 0 | 0 | 0 | 2 (9.5) ^(f)^ |
| Negative, n (%) | 379 (84.8) | 139 (100) | 271 (100) | 208 (100) | 63 (100) | 19 (90.5) |

SD, standard deviation

(a) TAK-003 was administered at Month 0 and Month 3 (without concomitant vaccination against yellow fever [DEN-305] or hepatitis A virus [DEN-314]).

(b) Based on the 3 TAK-003 lots combined.

(c) Two of the 21 flavivirus-naive subjects initially tested baseline seronegative (based on MNT_50_) were retrospectively tested baseline seropositive for DENV-1 upon repeat testing. Because enrolment of both subjects was performed according to the protocol, these subjects were not excluded from the PPS.

N listed in the table refer to the number of participants who were included in groups receiving TAK-003 in the respective studies

## Supplementary Table 2. Percentage of participants with tetravalent seropositivity (95% CI) for (a) studies performed in children and adolescents from endemic regions by baseline serostatus and (b) studies performed in adults from non-endemic regions (all baseline seronegative participants)

(a) Endemic regions

| **Study visit** | **Baseline seronegative** | | | **Baseline seropositive** | | |
| --- | --- | --- | --- | --- | --- | --- |
|  | **DEN-301** | **DEN-313** | **DEN-204** | **DEN-301** | **DEN-313** | **DEN-204** |
| **Baseline** | 0 (0.0, 0.5) | 0 (0.0, 4.2) | 0 (0.0, 8.4) | 83.5 (81.7, 85.2) | 67.9 (58.3, 76.5) | 80.5 (65.1, 91.2) |
| **Month 1** | 85.3 (82.4, 87.9) | 80.7 (70.6, 88.6) | 73.8 (58.0, 86.1) | 99.1 (98.5, 99.5) | 99.0 (94.7, 100) | 85.4 (70.8, 94.4) |
| **Month 3** | 84.3 (81.4, 86.9) | 68.6 (57.7, 78.2) | 64.3 (48.0, 78.4) | 98.6 (97.9, 99.1) | 95.4 (89.6, 98.5) | 95.1 (83.5, 99.4) |
| **Month 4** | 99.5 (98.6, 99.9) | 98.7 (93.1, 100) | – | 99.8 (99.5, 100) | 100 (96.5, 100) | – |
| **Month 6/9^(a)^** | 91.3 (88.7, 93.4) | 83.8 (73.8, 91.1) | 85.7 (71.5, 94.6) | 99.2 (98.7, 99.6) | 100 (96.6, 100) | 97.6 (87.1, 99.9) |
| **Month 15** | 99.1 (98.5, 99.5) | 73.8 (63.1, 82.8) | – | 87.8 (84.9, 90.3) | 98.1 (93.5, 99.8) | – |
| **Month 27** | 98.8 (98.1, 99.3) | 75.4 (62.7, 85.5) | – | 85.9 (82.4, 88.9) | 93.5 (86.5, 97.6) | – |
| **Month 39** | 98.3 (97.4, 98.9) | 55.1 (43.4, 66.4) | – | 80.3 (76.7, 83.6) | 93.1 (86.4, 97.2) | – |
| **Month 51** | 98.2 (97.4, 98.8) | – | – | 76.2 (72.4, 79.7) | – | – |

(b) Non-endemic regions

| **Study visit** | **DEN-304** | **DEN-307** | **DEN-315** | **DEN-305** | **DEN-314** | **DEN-210** |
| --- | --- | --- | --- | --- | --- | --- |
| **Baseline** | – | – | – | – | – | – |
| **Month 1** | – | – | – | 79.3 (72.9, 84.8) | 71.7 (58.6, 82.5) | 95.2 (76.2, 99.9) |
| **Month 4** | 95.6 (93.0, 97.5) | 97.0 (92.4, 99.2) | 99.6 (97.7, 100) | 97.0 (93.5, 98.9) | 90.9 (80.0, 97.0) | 91.7 (61.5, 99.8) |
| **Month 9** | 85.1 (80.9, 88.6) | 78.9 (71.0, 85.5) | 85.8 (80.9, 89.9) | – | – | 81.8 (48.2, 97.7) |

^(a)^ Measurements were performed at Month 9, with the exception of the DEN-204 study which was performed at Month 6

## Supplementary Table 3. Exploratory immunology assessments in children and adolescents in endemic regions and adults in non-endemic regions (baseline seronegative participants), by dengue serotype (9)

| **Measure** | **Time point** | **Region and age** | **DENV-1** | **DENV-2** | **DENV-3** | **DENV-4** |
| --- | --- | --- | --- | --- | --- | --- |
| **Dengue total binding IgG concentrations (RU/mL)** | Month 4 | Endemic, 4–16 years | 1,165.8 (937.6, 1,449.6) | 1,292.0 (1,060.7, 1,573.7) | 1,414.4 (1,180.2, 1,695.1) | 641.3 (528.5, 778.1) |
|  |  | Non-endemic, 18–60 years | 1,056.9 (826.3, 1,352.0) | 1,263.5 (1,005.8, 1,587.1) | 951.3 (774.4, 1,168.5) | 469.9 (370.5, 596.0) |
|  | Month 9 | 4–16 years | 735.2 (579.8, 932.2) | 894.3 (710.2, 1,126.1) | 835.4 (670.6, 1,040.8) | 465.4 (370.9, 583.9) |
|  |  | 18–60 years | 723.6 (544.7, 961.3) | 844.3 (653.2, 1,091.1) | 610.5 (486.4, 766.2) | 329.5 (257.3, 421.9) |
| **Anti-dengue IgG avidity (nm*s)** | Month 4 | 4–16 years | 526.7 (300.4, 923.6) | 424.2 (225.8, 796.7) | 307.2 (161.5, 584.6) | 147.3 (70.4, 307.8) |
|  |  | 18–60 years | 796.7 (433.3, 1,464.9) | 813.4 (466.5, 1,418.1) | 403.8 (217.5, 749.8) | 224.2 (105.4, 477.0) |
|  | Month 9 | 4–16 years | 222.8 (98.6, 503.8) | 315.0 (146.2, 679.0) | 92.8 (37.9, 227.3) | 35.1 (13.6, 91.1) |
|  |  | 18–60 years | 829.3 (477.7, 1,439.6) | 931.7 (629.8, 1,378.4) | 330.5 (163.7, 667.4) | 108.8 (42.6, 278.0) |
| **Anti-dengue NS1 IgG concentration (EU/mL)** | Month 4 | Endemic, 4–16 years | 245.7 (213.0, 283.6) | 1,053.0 (882.2, 1,256.8) | 219.2 (189.8, 253.3) | 178.7 (149.5, 213.7) |
|  |  | Non-endemic, 18–60 years | 299.2 (232.8, 384.4) | 1,299.9 (1,032.1, 1,637.1) | 210.3 (157.8, 280.4) | 144.1 (106.0, 196.0) |
|  | Month 9 | Endemic, 4–16 years | 140.8 (118.3, 167.7) | 365.5 (303.4, 440.2) | 101.4 (88.3, 116.4) | 85.8 (73.0, 100.8) |
|  |  | Non-endemic, 18–60 years | 185.3 (146.6, 234.2) | 624.5 (494.3, 789.0) | 114.3 (87.6, 149.2) | 83.8 (64.1, 109.7) |
| **Anti-dengue complement antibody titres (EU/mL)** | Month 4 | 4–16 years | 36.8 (27.9, 48.7) | 31.6 (24.4, 40.8) | 33.6 (25.7, 43.9) | 12.7 (9.5, 16.9) |
|  |  | 18–60 years | 25.3 (18.8, 34.0) | 24.5 (19.0, 31.7) | 19.3 (14.3, 26.1) | 9.6 (7.3, 12.6) |
|  | Month 9 | 4–16 years | 17.1 (11.6, 25.0) | 15.3 (10.8, 21.6) | 13.3 (9.0, 19.8) | 6.4 (4.5, 8.9) |
|  |  | 18–60 years | 14.7 (10.4, 20.8) | 13.1 (9.5, 18.1) | 9.6 (6.8, 13.6) | 5.9 (4.3, 8.0) |
| **Percent of type-specific neutralising antibody response/EC_50_** **(RVP) after DENV-2 NAb depletion (%)** | Month 4 | 4–16 years | 47.4 (31.0, 64.2) | DeMaso et al. (39) | 57.1 (39.4, 73.7) | 94.4 (81.3, 99.3) |
|  |  | 18–60 years | 68.0 (46.5, 85.1) | DeMaso et al. (39) | 72.2 (46.5, 90.3) | 91.3 (72.0, 98.9) |
|  | Month 9 | 4–16 years | 20.0 (5.7, 43.7) | DeMaso et al. (39) | 47.1 (23.0, 72.2) | 100.0 (83.2, 100.0) |
|  |  | 18–60 years | 76.2 (52.8, 91.8) | DeMaso et al. (39) | 37.5 (15.2, 64.6) | 94.4 (72.7, 99.9) |

CI, confidence interval; DENV, dengue virus; EC_50_, 50% effective concentration; EU/mL, effective units per millilitre; IgG, immunoglobulin G; NAb, neutralising antibody; NS1, non-structural protein 1; RU/mL, relative units per millilitre; RVP, reporter virus particle. Summary of exploratory immunology assessments and readouts for each serotype in participants aged 4–16 years (DEN-301) vs. aged 18–60 years (DEN-304). Data are presented as geometric means (95% CIs) unless otherwise stated. A total of 48 participants were included in the analysis in each study.

## Supplementary Table 4. Hazard ratios (95% CI) of correlate of risk of VCD analysis between 1 month after second dose and until 18 months after the second dose (approximately Month 21 after the first dose; per protocol set – correlate of protection subset data) and by baseline serostatus based on neutralising antibody titres one month after the second dose (Month 4) (23)

|  | Medium vs Low Titre ^(a)^ | High vs Low Titre ^(a)^ |
| --- | --- | --- |
| Serotype | TAK-003 | TAK-003 |
| **Overall** |  |  |
| **DENV-1** | 0.507 (0.259, 0.993) | 0 (0,0) |
| **DENV-2** | NE (NE, NE) | NE (NE, NE) |
| **DENV-3** | 0.543 (0.321, 0.918) | 0.024 (0.003, 0.181) |
| **DENV-4** | 0.537 (0.071, 4.056) | 0 (0,0) |
| **Baseline seropositive** |  |  |
| **DENV-1** | 0.445 (0.171, 1.156) | 0 (0,0) |
| **DENV-2** | 0.398 (0.043, 3.720) | 0.169 (0.016, 1.819) |
| **DENV-3** | 0.217 (0.102, 0.462) | 0 (0,0) |
| **DENV-4** | 0.393 (0.031, 4.931) | 0 (0,0) |
| **Baseline seronegative** |  |  |
| **DENV-1** | NE (NE, NE) | NE (NE, NE) |
| **DENV-2** | 0.398 (0.043, 3.720) | 0.169 (0.016, 1.819) |
| **DENV-3** | 0.483 (0.098, 2.381) | 0.288 (0.054, 1.542) |
| **DENV-4** | NE (NE, NE) | 1.033 (0.136, 7.839) |

CI, confidence interval; NE, non-estimable.

(a)	Low, medium, and high titre subgroups defined by weighted tertiles of Month 4 titres pooling over the 4 serotypes and over the TAK-003 and placebo groups. The weights are defined to account for the case-cohort biomarkers sampling design.

Hazard ratio (λV / λC) and 2-sided 95% CI estimated from the Cox proportional hazards regression models for each treatment group including the serotype-specific geometric mean titre as covariate adjusting for age and stratified by region. If there were no cases in the medium or high tertiles compared with the low tertile, the hazard ratio was 0. If there no cases in the low tertile, the hazard ratio was non-estimable. A greater than 1 hazard ratio is in favour of low tertile group in the comparison, a less than 1 hazard ratio is in favour of medium or high tertile group, within either placebo group or TAK-003 group.

**References – Supplementary Materials**

1.	Mandaric S, Friberg H, Saez-Llorens X, Borja-Tabora C, Biswal S, Escudero I, et al. Long Term T Cell Response and Safety of a Tetravalent Dengue Vaccine in Healthy Children. *NPJ Vaccines* (2024) 9(1):192. Epub 20241017. doi: 10.1038/s41541-024-00967-0.

2.	Tricou V, Sáez-Llorens X, Yu D, Rivera L, Jimeno J, Villarreal AC, et al. Safety and Immunogenicity of a Tetravalent Dengue Vaccine in Children Aged 2-17 Years: A Randomised, Placebo-Controlled, Phase 2 Trial. *Lancet* (2020) 395(10234):1434-43. Epub 2020/03/21. doi: 10.1016/s0140-6736(20)30556-0.

3.	Mandaric S, Friberg H, Gottardo R, Messer W, Tricou V, Faccin A, et al. Humoral and Cellular Responses to a Tetravalent Dengue Vaccine (Tak-003) in Adults from a Dengue Non-Endemic Region: An Open-Label Phase 2 Trial. *Vaccine* (2026) 77:128373. Epub 20260220. doi: 10.1016/j.vaccine.2026.128373.

4.	Tricou V, Winkle PJ, Tharenos LM, Rauscher M, Escudero I, Hoffman E, et al. Consistency of Immunogenicity in Three Consecutive Lots of a Tetravalent Dengue Vaccine Candidate (Tak-003): A Randomized Placebo-Controlled Trial in Us Adults. *Vaccine* (2023) 41(47):6999-7006. Epub 2023/10/27. doi: 10.1016/j.vaccine.2023.09.049.

5.	Tricou V, Essink B, Ervin JE, Turner M, Escudero I, Rauscher M, et al. Immunogenicity and Safety of Concomitant and Sequential Administration of Yellow Fever Yf-17d Vaccine and Tetravalent Dengue Vaccine Candidate Tak-003: A Phase 3 Randomized, Controlled Study. *PLoS Negl Trop Dis* (2023) 17(3):e0011124. Epub 2023/03/09. doi: 10.1371/journal.pntd.0011124.

6.	Patel SS, Winkle P, Faccin A, Nordio F, LeFevre I, Tsoukas CG. An Open-Label, Phase 3 Trial of Tak-003, a Live Attenuated Dengue Tetravalent Vaccine, in Healthy Us Adults: Immunogenicity and Safety When Administered during the Second Half of a 24-Month Shelf-Life. *Hum Vaccin Immunother* (2023) 19(2):2254964. Epub 2023/10/17. doi: 10.1080/21645515.2023.2254964.

7.	Tricou V, Eyre S, Ramjee M, Collini P, Mojares Z, Loeliger E, et al. A Randomized Phase 3 Trial of the Immunogenicity and Safety of Coadministration of a Live-Attenuated Tetravalent Dengue Vaccine (Tak-003) and an Inactivated Hepatitis a (Hav) Virus Vaccine in a Dengue Non-Endemic Country. *Vaccine* (2023) 41(7):1398-407. Epub 2023/01/22. doi: 10.1016/j.vaccine.2023.01.007.

8.	Biswal S, Mendez Galvan JF, Macias Parra M, Galan-Herrera JF, Carrascal Rodriguez MB, Rodriguez Bueno EP, et al. Immunogenicity and Safety of a Tetravalent Dengue Vaccine in Dengue-Naïve Adolescents in Mexico City. *Rev Panam Salud Publica* (2021) 45:e67. Epub 2021/06/17. doi: 10.26633/rpsp.2021.67.

9.	LeFevre I, Bravo L, Folschweiller N, Medina EL, Moreira ED, Jr., Nordio F, et al. Bridging the Immunogenicity of a Tetravalent Dengue Vaccine (Tak-003) from Children and Adolescents to Adults. *NPJ Vaccines* (2023) 8(1):75. Epub 2023/05/26. doi: 10.1038/s41541-023-00670-6.

10.	Nascimento EJM, Norwood B, Kpamegan E, Parker A, Fernandes J, Perez-Guzman E, et al. Antibodies Produced in Response to a Live-Attenuated Dengue Vaccine Are Functional in Activating the Complement System. *J Infect Dis* (2023) 227(11):1282-92. Epub 2022/12/04. doi: 10.1093/infdis/jiac476.

11.	Rupp R, Luckasen GJ, Kirstein JL, Osorio JE, Santangelo JD, Raanan M, et al. Safety and Immunogenicity of Different Doses and Schedules of a Live Attenuated Tetravalent Dengue Vaccine (Tdv) in Healthy Adults: A Phase 1b Randomized Study. *Vaccine* (2015) 33(46):6351-9. Epub 20150915. doi: 10.1016/j.vaccine.2015.09.008.

12.	Sharma M, Glasner DR, Watkins H, Puerta-Guardo H, Kassa Y, Egan MA, et al. Magnitude and Functionality of the Ns1-Specific Antibody Response Elicited by a Live-Attenuated Tetravalent Dengue Vaccine Candidate. *J Infect Dis* (2020) 221(6):867-77. Epub 2019/02/21. doi: 10.1093/infdis/jiz081.

13.	Sirivichayakul C, Barranco-Santana EA, Rivera IE, Kilbury J, Raanan M, Borkowski A, et al. Long-Term Safety and Immunogenicity of a Tetravalent Dengue Vaccine Candidate in Children and Adults: A Randomized, Placebo-Controlled, Phase 2 Study. *J Infect Dis* (2022) 225(9):1513-20. Epub 2020/07/14. doi: 10.1093/infdis/jiaa406.

14.	Tsuji I, Dominguez D, Egan MA, Dean HJ. Development of a Novel Assay to Assess the Avidity of Dengue Virus-Specific Antibodies Elicited in Response to a Tetravalent Dengue Vaccine. *J Infect Dis* (2022) 225(9):1533-44. doi: 10.1093/infdis/jiab064.

15.	Sáez-Llorens X, Tricou V, Yu D, Rivera L, Jimeno J, Villarreal AC, et al. Immunogenicity and Safety of One Versus Two Doses of Tetravalent Dengue Vaccine in Healthy Children Aged 2-17 Years in Asia and Latin America: 18-Month Interim Data from a Phase 2, Randomised, Placebo-Controlled Study. *Lancet Infect Dis* (2018) 18(2):162-70. Epub 20171106. doi: 10.1016/s1473-3099(17)30632-1.

16.	Sáez-Llorens X, Tricou V, Yu D, Rivera L, Tuboi S, Garbes P, et al. Safety and Immunogenicity of One Versus Two Doses of Takeda's Tetravalent Dengue Vaccine in Children in Asia and Latin America: Interim Results from a Phase 2, Randomised, Placebo-Controlled Study. *Lancet Infect Dis* (2017) 17(6):615-25. Epub 20170330. doi: 10.1016/s1473-3099(17)30166-4.

17.	Tricou V, Gottardo R, Egan MA, Clement F, Leroux-Roels G, Sáez-Llorens X, et al. Characterization of the Cell-Mediated Immune Response to Takeda's Live-Attenuated Tetravalent Dengue Vaccine in Adolescents Participating in a Phase 2 Randomized Controlled Trial Conducted in a Dengue-Endemic Setting. *Vaccine* (2022) 40(8):1143-51. Epub 2022/01/27. doi: 10.1016/j.vaccine.2022.01.016.

18.	Michlmayr D, Andrade P, Nascimento EJM, Parker A, Narvekar P, Dean HJ, et al. Characterization of the Type-Specific and Cross-Reactive B-Cell Responses Elicited by a Live-Attenuated Tetravalent Dengue Vaccine. *J Infect Dis* (2021) 223(2):247-57. doi: 10.1093/infdis/jiaa346.

19.	Tricou V, Low JG, Oh HM, Leo YS, Kalimuddin S, Wijaya L, et al. Safety and Immunogenicity of a Single Dose of a Tetravalent Dengue Vaccine with Two Different Serotype-2 Potencies in Adults in Singapore: A Phase 2, Double-Blind, Randomised, Controlled Trial. *Vaccine* (2020) 38(6):1513-9. Epub 2019/12/18. doi: 10.1016/j.vaccine.2019.11.061.

20.	Biswal S, Borja-Tabora C, Martinez Vargas L, Velásquez H, Theresa Alera M, Sierra V, et al. Efficacy of a Tetravalent Dengue Vaccine in Healthy Children Aged 4-16 Years: A Randomised, Placebo-Controlled, Phase 3 Trial. *Lancet* (2020) 395(10234):1423-33. Epub 2020/03/21. doi: 10.1016/s0140-6736(20)30414-1.

21.	Biswal S, Reynales H, Saez-Llorens X, Lopez P, Borja-Tabora C, Kosalaraksa P, et al. Efficacy of a Tetravalent Dengue Vaccine in Healthy Children and Adolescents. *N Engl J Med* (2019) 381(21):2009-19. Epub 2019/11/07. doi: 10.1056/NEJMoa1903869.

22.	López-Medina E, Biswal S, Saez-Llorens X, Borja-Tabora C, Bravo L, Sirivichayakul C, et al. Efficacy of a Dengue Vaccine Candidate (Tak-003) in Healthy Children and Adolescents 2 Years after Vaccination. *J Infect Dis* (2022) 225(9):1521-32. Epub 2020/12/16. doi: 10.1093/infdis/jiaa761.

23.	Rivera L, Biswal S, Sáez-Llorens X, Reynales H, López-Medina E, Borja-Tabora C, et al. Three-Year Efficacy and Safety of Takeda's Dengue Vaccine Candidate (Tak-003). *Clin Infect Dis* (2022) 75(1):107-17. Epub 2021/10/05. doi: 10.1093/cid/ciab864.

24.	Tricou V, Yu D, Reynales H, Biswal S, Saez-Llorens X, Sirivichayakul C, et al. Long-Term Efficacy and Safety of a Tetravalent Dengue Vaccine (Tak-003): 4·5-Year Results from a Phase 3, Randomised, Double-Blind, Placebo-Controlled Trial. *Lancet Glob Health* (2024) 12(2):e257-e70. doi: 10.1016/s2214-109x(23)00522-3.

25.	Borja-Tabora C, Fernando L, Lopez Medina E, Reynales H, Rivera L, Saez-Llorens X, et al. Immunogenicity, Safety, and Efficacy of a Tetravalent Dengue Vaccine in Children and Adolescents: An Analysis by Age Group. *Clin Infect Dis* (2025) 80(1):199-206. doi: 10.1093/cid/ciae369.

26.	Sáez-Llorens X, Biswal S, Borja-Tabora C, Fernando L, Liu M, Wallace D, et al. Effect of the Tetravalent Dengue Vaccine Tak-003 on Sequential Episodes of Symptomatic Dengue. *Am J Trop Med Hyg* (2023) 108(4):722-6. Epub 2023/03/07. doi: 10.4269/ajtmh.22-0673.

27.	Sirivichayakul C, Biswal S, Saez-Llorens X, López-Medina E, Borja-Tabora C, Bravo L, et al. Efficacy and Safety of a Tetravalent Dengue Vaccine (Tak-003) in Children with Prior Japanese Encephalitis or Yellow Fever Vaccination. *J Infect Dis* (2024) 230(6):e1214-e25. doi: 10.1093/infdis/jiae222.

28.	Sharma M, Nascimento EJM, DeMaso CR. Diversity, Quality, Magnitude and Functionality of the Humoral Response to a Live-Attenuated Tetravelent Dengue Vaccine in Pediatric Participants from a Phase 3 Efficacy Trial. *41st Annual Meeting of the European Society for Paediatric Infectious Diseases*; 8-12 May; Lisbon, Portugal(2023).

29.	El Hindi T, Anugulruengkitt S, Lapphra K, Limkittikul K, Tangsathapornpong A, Galindo-Tsoukas C, et al. Immunogenicity and Safety of the Live-Attenuated Tetravalent Dengue Vaccine (Tak-003) Co-Administered with Recombinant 9-Valent Human Papillomavirus Vaccine. *Vaccine* (2025) 62:127558. Epub 20250731. doi: 10.1016/j.vaccine.2025.127558.

30.	Chu H, George SL, Stinchcomb DT, Osorio JE, Partidos CD. Cd8+ T-Cell Responses in Flavivirus-Naive Individuals Following Immunization with a Live-Attenuated Tetravalent Dengue Vaccine Candidate. *J Infect Dis* (2015) 212(10):1618-28. Epub 2015/05/07. doi: 10.1093/infdis/jiv258.

31.	George SL, Wong MA, Dube TJ, Boroughs KL, Stovall JL, Luy BE, et al. Safety and Immunogenicity of a Live Attenuated Tetravalent Dengue Vaccine Candidate in Flavivirus-Naive Adults: A Randomized, Double-Blinded Phase 1 Clinical Trial. *J Infect Dis* (2015) 212(7):1032-41. Epub 20150319. doi: 10.1093/infdis/jiv179.

32.	Kim EY, Che Y, Dean HJ, Lorenzo-Redondo R, Stewart M, Keller CK, et al. Transcriptome-Wide Changes in Gene Expression, Splicing, and Lncrnas in Response to a Live Attenuated Dengue Virus Vaccine. *Cell Rep* (2022) 38(6):110341. doi: 10.1016/j.celrep.2022.110341.

33.	Osorio JE, Velez ID, Thomson C, Lopez L, Jimenez A, Haller AA, et al. Safety and Immunogenicity of a Recombinant Live Attenuated Tetravalent Dengue Vaccine (Denvax) in Flavivirus-Naive Healthy Adults in Colombia: A Randomised, Placebo-Controlled, Phase 1 Study. *Lancet Infect Dis* (2014) 14(9):830-8. Epub 20140723. doi: 10.1016/s1473-3099(14)70811-4.

34.	Jackson LA, Rupp R, Papadimitriou A, Wallace D, Raanan M, Moss KJ. A Phase 1 Study of Safety and Immunogenicity Following Intradermal Administration of a Tetravalent Dengue Vaccine Candidate. *Vaccine* (2018) 36(27):3976-83. Epub 20180519. doi: 10.1016/j.vaccine.2018.05.028.

35.	Swanstrom JA, Henein S, Plante JA, Yount BL, Widman DG, Gallichotte EN, et al. Analyzing the Human Serum Antibody Responses to a Live Attenuated Tetravalent Dengue Vaccine Candidate. *J Infect Dis* (2018) 217(12):1932-41. doi: 10.1093/infdis/jiy063.

36.	Waickman AT, Friberg H, Gargulak M, Kong A, Polhemus M, Endy T, et al. Assessing the Diversity and Stability of Cellular Immunity Generated in Response to the Candidate Live-Attenuated Dengue Virus Vaccine Tak-003. *Front Immunol* (2019) 10:1778. Epub 2019/08/17. doi: 10.3389/fimmu.2019.01778.

37.	Waickman AT, Victor K, Li T, Hatch K, Rutvisuttinunt W, Medin C, et al. Dissecting the Heterogeneity of Denv Vaccine-Elicited Cellular Immunity Using Single-Cell Rna Sequencing and Metabolic Profiling. *Nat Commun* (2019) 10(1):3666. Epub 2019/08/16. doi: 10.1038/s41467-019-11634-7.

38.	Turner M, Papadimitriou A, Winkle P, Segall N, Levin M, Doust M, et al. Immunogenicity and Safety of Lyophilized and Liquid Dengue Tetravalent Vaccine Candidate Formulations in Healthy Adults: A Randomized, Phase 2 Clinical Trial. *Hum Vaccin Immunother* (2020) 16(10):2456-64. Epub 2020/03/03. doi: 10.1080/21645515.2020.1727697.

39.	DeMaso CR, Karwal L, Zahralban-Steele M, Dominguez D, Springer ZL, Kaiser M, et al. Specificity and Breadth of the Neutralizing Antibody Response to a Live-Attenuated Tetravalent Dengue Vaccine. *J Infect Dis* (2022) 226(11):1959-63. doi: 10.1093/infdis/jiac272.
